# Perceptual-Motor and Perceptual-Cognitive Skill Acquisition in Soccer: A Systematic Review on the Influence of Practice Design and Coaching Behavior

**DOI:** 10.3389/fpsyg.2021.772201

**Published:** 2021-12-02

**Authors:** Fynn Bergmann, Rob Gray, Svenja Wachsmuth, Oliver Höner

**Affiliations:** ^1^Institute of Sports Science, Eberhard Karls University Tübingen, Tübingen, Germany; ^2^Human Systems Engineering, Arizona State University, Mesa, AZ, United States

**Keywords:** football (soccer), talent development, ecological dynamics, dynamical systems, information-processing

## Abstract

Facilitating players' skill acquisition is a major challenge within sport coaches' work which should be supported by evidence-based recommendations outlining the most effective practice and coaching methods. This systematic review aimed at accumulating empirical knowledge on the influence of practice design and coaching behavior on perceptual-motor and perceptual-cognitive skill acquisition in soccer. A systematic search was carried out according to the PRISMA guidelines across the databases SPORTDiscus, PsycInfo, MEDLINE, and Web of Science to identify soccer-specific intervention studies conducted in applied experimental settings (search date: 22^nd^ November 2020). The systematic search yielded 8,295 distinct hits which underwent an independent screening process. Finally, 34 eligible articles, comprising of 35 individual studies, were identified and reviewed regarding their theoretical frameworks, methodological approaches and quality, as well as the interventions' effectiveness. These studies were classified into the following two groups: Eighteen studies investigated the theory-driven instructional approaches Differential Learning, Teaching Games for Understanding, and Non-linear Pedagogy. Another seventeen studies, most of them not grounded within a theoretical framework, examined specific aspects of practice task design or coaches' instructions. The Downs and Black checklist and the Template for Intervention Description and Replication were applied to assess the quality in reporting, risk of bias, and the quality of interventions' description. Based on these assessments, the included research was of moderate quality, however, with large differences across individual studies. The quantitative synthesis of results revealed empirical support for the effectiveness of coaching methodologies aiming at encouraging players' self-exploration within representative scenarios to promote technical and tactical skills. Nevertheless, “traditional” repetition-based approaches also achieved improvements with respect to players' technical outcomes, yet, their impact on match-play performance remains widely unexplored. In the light of the large methodological heterogeneity of the included studies (e.g., outcomes or control groups' practice activities), the presented results need to be interpreted by taking the respective intervention characteristics into account. Overall, the current evidence needs to be extended by theory-driven, high-quality studies within controlled experimental designs to allow more consolidated and evidence-based recommendations for coaches' work.

## Introduction

Sport coaches face multiple challenges, one of which is to facilitate athletes' skill acquisition to improve performance (Gould and Mallett, 2021). Due to the dynamic and interactive character of team sports games, a plethora of skills is required to act successfully during gameplay. In soccer, these skills specifically encompass perceptual-motor (e.g., technical) and perceptual-cognitive (e.g., tactical) components (Williams et al., 2020). Knowledge about these and further relevant performance factors is not only important to identify talented players, it is also essential for developing these performance factors systematically through effective practice and coaching. While recent systematic reviews document the growing body of knowledge about soccer-specific performance characteristics and talent predictors, less is known on how to promote these factors effectively, reinforcing the call for intervention research (Williams et al., 2020; O'Connor et al., 2021).

Most intervention research on the development of performance factors in soccer is concerned with the players' physical fitness and physiological capabilities (for overviews see Bujalance-Moreno et al., 2018; Zouhal et al., 2020). Considerably fewer studies investigated the promotion of soccer-specific skills based on psychological and motor learning theories (Williams and Hodges, 2005). This smaller scope of scientific work is primarily attributed to methodological challenges in assessing behavioral changes in players, especially in highly dynamic and unpredictable match-play situations. For this reason, laboratory-based research on the acquisition of closed soccer skills has made the primary contribution to our general understanding of skill acquisition (e.g., Anderson and Sidaway, 1994; Hodges et al., 2005). Often conducted with novice participants, those studies seem to not only lack transferability to a pitch-based coaching context but also to superior skill-level players (Wulf and Shea, 2002; Farrow and Robertson, 2017). As a possible consequence, there is an emerging trend toward intervention studies anchored in more representative settings and with more experienced players (e.g., Práxedes et al., 2016; Roberts et al., 2020).

### Theoretical Perspectives on Skill Acquisition

Besides methodological difficulties in designing intervention research, also the theoretical underpinnings of skill acquisition pose challenges for developing effective practice. There are different perspectives on the organization and development of skills, possibly leading to contradictory implications for coaches' work. Historically, substantial advances have been made with regards to understanding the processes linked to skill acquisition, but it remains a dynamic and disputed topic among researchers (Whitall et al., 2020a,b).

In current research, two influential theoretical perspectives can be distinguished that emerged from cognitive psychology, on the one hand, and ecological psychology/dynamical systems theory, on the other hand (Anson et al., 2005).^1^ According to an understanding grounded in *cognitive “information-processing”* theories, there is an ideal way to perform a skill that is to be learned through a stage-linear process (Fitts and Posner, 1979). Based on the schema theory of motor learning (Schmidt, 1975), a skill consists of invariants that are stored through mental representations in so-called generalized motor programs (GMPs). For utilizing GMPs in dynamic game-related situations, players must learn to parameterize the skill, that means, learn how to adjust the skill to the requirements of any respective situation. Within this theoretical perspective, performing a game action relates to a subsequent three-stage process from stimulus identification (i.e., perception), response selection (i.e., decision), and response programming (i.e., action; Schmidt et al., 2019).

A different, opposing view to this often called “traditional” standpoint emerges from an *ecological/dynamical systems* perspective where skill acquisition is considered a process of exploration and self-organization. Based on Newell's (1986) constraints-based model, individuals interact with the environment and the tasks of the given situation by exploring individual movement solutions in a non-linear fashion. In this approach, there is no one ideal, “correct” technique for performing a skill. Rather, there is substantial variability both across and within individuals (i.e., “repetition without repetition”; Bernstein, 1967). According to this viewpoint, performing game actions depends on the direct perception of affordances from the environment, implying that perception, and action are considered inherently coupled (Gibson, 1979).

### Practice and Coaching Methods to Promote Skill Acquisition in Soccer

In searching for the most supportive methods to improve players' skills, it is worthwhile to look at the early stages of playing soccer during childhood: In these stages, the process of skill acquisition can occur in an implicit and unstructured way within informal and child-led settings, such as street soccer (Uehara et al., 2018). There is evidence that a high amount of such self-organized soccer gameplays, as well as multi-sport practice in childhood, is positively associated with achieving excellence in adult soccer (Forsman et al., 2016; Güllich et al., 2021). However, when looking at the pathways of elite players, it is indisputable that formal soccer-specific and coach-led practice is indispensable to improve and sustain players' skill level, too (Sieghartsleitner et al., 2018; Hendry and Hodges, 2019). In this goal-directed process, coaches are challenged by a variety of methodological decisions relating to the skill itself but also contextual factors, such as the athlete's performance level, age, or available time to achieve an outcome (Côté and Gilbert, 2009). Therefore, coaches likely need a “blended tool kit” (Price et al., 2019, p. 126), equipped with practice and coaching methods in order to find effective context-specific solutions to facilitate player learning and performance.

To describe these “tools”, the terms *training form* (i.e., decontextualized repetitive drills) and *playing form* (i.e., game-related, representative situations) are commonly used to classify practice activities in soccer (Ford et al., 2010; O'Connor et al., 2018). Yet, these terms only provide a broad picture of the accompanying task demands so that more sophisticated differentiation is needed. One of that is the degree of variability within (i.e., trial-to-trial) and between (i.e., contextual interference) practiced skills that can be scaled in drills and game-based tasks (Stratton et al., 2004; Magill and Anderson, 2020). Further, the specificity describes whether practice conditions reflect competitive demands in terms of motor- and sensory-related parameters (Proteau, 1992; Farrow and Robertson, 2017). Besides practice activities, there is also a wide array of coaching behaviors, such as demonstrations, instructions, and feedback which are used to accentuate the practice demands (Hendry et al., 2015; Otte et al., 2020). For instance, instructive behaviors may differ depending on whether explicit or implicit learning processes should be promoted in the players.

Insights to which extent coaches make use of the outlined tools during pitch-based work are given by systematic observations in “real-world” coaching contexts (Cushion et al., 2012a; Partington and Cushion, 2013; Partington et al., 2014). Many of these studies have displayed the “challenging tradition” of practice, instruction, and skill acquisition in terms of the predominant use of unrepresentative drill-based activities and a high amount of prescriptive instructions by the coaches (Williams and Hodges, 2005). More recent studies show a trend regarding more game-realistic activities which are more closely aligned to competitive demands (O'Connor et al., 2018; Roca and Ford, 2020). Nonetheless, there still seems to be potential toward greater consideration of skill acquisition research within soccer coaches' work (O'Connor et al., 2017; Farrow, 2021).

In contrast to the outlined traditional approach, characterized by the predominant use of decontextualized drill-based activities and directive instructions, instructional approaches providing alternative strategies have gained increasing popularity in soccer. Grounded in an educative and constructivist perspective, *Teaching Games for Understanding* (TGFU; Bunker and Thorpe, 1982) is a game-centered approach that aims to improve tactical intelligence through simplified, game-related situations. The accompanying guided discovery approach to coaching is intended to explicitly enhance players in solving tactical problems. As another approach, *Non-linear Pedagogy* (NLP; Chow et al., 2011) provides key principles to practice and coaching and is shaped by an ecological dynamics viewpoint. According to NLP, functional variability of skills can be achieved through perception-action couplings during practice, by applying representative learning designs, and a “hands-off” facilitative approach to coaching. NLP aims to support players in finding individual movement solutions through a non-linear process of learning. Lastly, based on dynamical systems theory, *Differential Learning* (DL; Schöllhorn, 1999) assumes that athletes need to adapt to constant perturbations in dynamic competitive environments. Thus, practicing skills with additional random fluctuations (“noise”) offers the opportunity to explore and self-organize individual functional movement patterns.

Considering the wide array of possible practice and coaching approaches, Williams et al. (2020) and O'Connor et al. (2021) call for intervention research on soccer players' skill acquisition to get a deeper insight into the effectiveness of different methods. Recent systematic reviews and meta-analyses within these and related fields pooled intervention research from educational settings (Abad Robles et al., 2020), focused on the effectiveness of one instructional approach (e.g., DL; Tassignon et al., 2021), or merely examined one specific outcome (e.g., decision-making; Silva et al., 2020). While these reviews included studies from various sports and often different experimental settings, there is no systematic review accumulating evidence on the effectiveness of practice and coaching methods grounded in different theoretical perspectives to skill acquisition from a soccer-specific viewpoint.

### The Present Study

This systematic review aims to pool empirical knowledge from intervention research on the effectiveness of different practice and coaching methods on skill acquisition in soccer. In contrast to previous systematic reviews and meta-analyses, the current review is explicitly set out to focus on the following attributes: First, it focuses on intervention research conducted in applied (“pitch-based”) experimental settings which included experienced soccer players as participants rather than novices. Such studies provide the ecological validity necessary to offer the most pertinent support for soccer coaches' actual work. Next, it was deemed vital to investigate studies grounded in different theoretical skill acquisition approaches to explore how different assumptions impact an interventions' design as well as to discuss the effectiveness of different approaches regarding the acquisition and learning of soccer-specific skills. Finally, considering both perceptual-motor and perceptual-cognitive skills as outcomes was perceived mandatory due to their interrelationship during matchplay. As there is no overview of intervention research in soccer considering these aspects, knowledge on the methodological characteristics and rigor is required to estimate the current potential for drawing evidence-based recommendations for coaches' work.

To this end, soccer-specific intervention studies conducted in applied experimental settings with soccer players were reviewed to answer the following research questions considering three overarching perspectives:

- *Theoretical perspective:* What were the interventions' underlying frameworks to skill acquisition? How did these impact the practice design and coaches' behavior?
- M*ethodological perspective:* What study designs, participant samples, instruments, and statistical methods were applied? What was the quality in reporting and risk of bias within studies?
- *Outcome-related perspective:* To what degree did the interventions contribute to effective perceptual-motor and perceptual-cognitive skill acquisition in the players?

## Methods

A systematic review was conducted according to the guidelines of preferred reporting items for systematic review and meta-analyses (PRISMA; Moher et al., 2009). On November 4^th^ 2020, a PRISMA-Protocol (PRISMA-P; Moher et al., 2015) was pre-registered in the Open Science Framework, outlining the objectives of this systematic review, the systematic search strategy, methods for assessing the methodological quality of individual studies, as well as predetermined methods for data extraction and synthesis in detail (Bergmann et al., 2020).

### Systematic Search and Eligibility Criteria

On November 22^nd^ 2020, a systematic search was conducted across the databases SPORTDiscus, PsycInfo, MEDLINE (via EBSCOHost, respectively), and Web of Science (considering the categories Sports Science and Psychology). Each database was searched for peer-reviewed academic articles in English language without limitations for publication year. The systematic search strategy was developed by the research group in consultation with two librarians who helped to optimize search terms and to identify the most appropriate databases to best address the objectives of this systematic review (Harari et al., 2020). The following terms and operators were searched in each database considering titles and keywords (further details of the systematic database search and the respective settings of each database are documented within Supplementary Material I):

(football^*^ OR soccer)
AND
(intervention OR train^*^ OR program^*^ OR approach OR pract^*^ OR effect^*^ OR impact OR improv^*^ OR learn^*^ OR perform^*^ OR coach^*^ OR “skill acquisition” OR cognit^*^ OR ecologic^*^ OR constraints OR “information processing”)
NOT
(novice OR referee OR injur^*^ OR pupil^*^ OR class OR goalkeep^*^ OR NFL OR “american football” OR “australian football”)

The inclusion criteria for study selection are presented in Table 1 according to the PICOS components. In accordance with Spittle's (2021) classification of intervention research, only studies in at least applied settings were included. Additionally, studies were excluded if they examined novices, goalkeepers, referees, or athletes with mental or physical disabilities. Besides that, interventions focusing on injury prevention or rehabilitation (including warm-up programs), as well as interventions that only included physical or physiological training exercises without a ball (i.e., fitness training), were not considered. This was also true for such studies which only assessed physical or physiological outcomes. Due to the lower ecological validity, laboratory research designs, but also imagery or virtual-reality interventions, were excluded.

**Table 1:** Specification of inclusion criteria regarding the components participants, interventions, comparators, outcomes, and study designs (PICOS).

**Component**	**Inclusion criteria**
P	• Healthy and injury-free soccer players (i.e., with previous soccer experience) of all age groups and sexes.
I	• Soccer-specific interventions conducted in applied (i.e., pitch-based) settings (cf. Spittle, 2021, p. 10).
C	• Studies without CG were included for review but only analyzed regarding theoretical frameworks as well as methodological characteristics and quality (i.e., perspectives I and II). In terms of the interventions' effectiveness (i.e., perspective III), only controlled designs including non-active and/or active control groups were considered.
O	• Soccer-specific perceptual-motor and/or perceptual-cognitive skills.
S	• Any type of quantitative (i.e., quasi-experimental and experimental) intervention study investigating the effectiveness of practice and coaching methods with regard to the acquisition or learning of the aforementioned soccer-specific skills.

*CG, control group*.

### Article Screening

The search results were exported to and managed with EndNote (Version X9.3). EndNote was also used to remove duplicates automatically. Additionally, the first author and a trained research assistant screened all titles independently to remove previously missed duplicates manually. Throughout an independent screening process following the PRISMA guidelines, potentially eligible articles were checked against the inclusion and exclusion criteria by both reviewers. The inter-rater agreement (IRA) was calculated using the percentage of agreement as well as Cohen's Kappa (κ; Hallgren, 2012).

**Figure 1:**
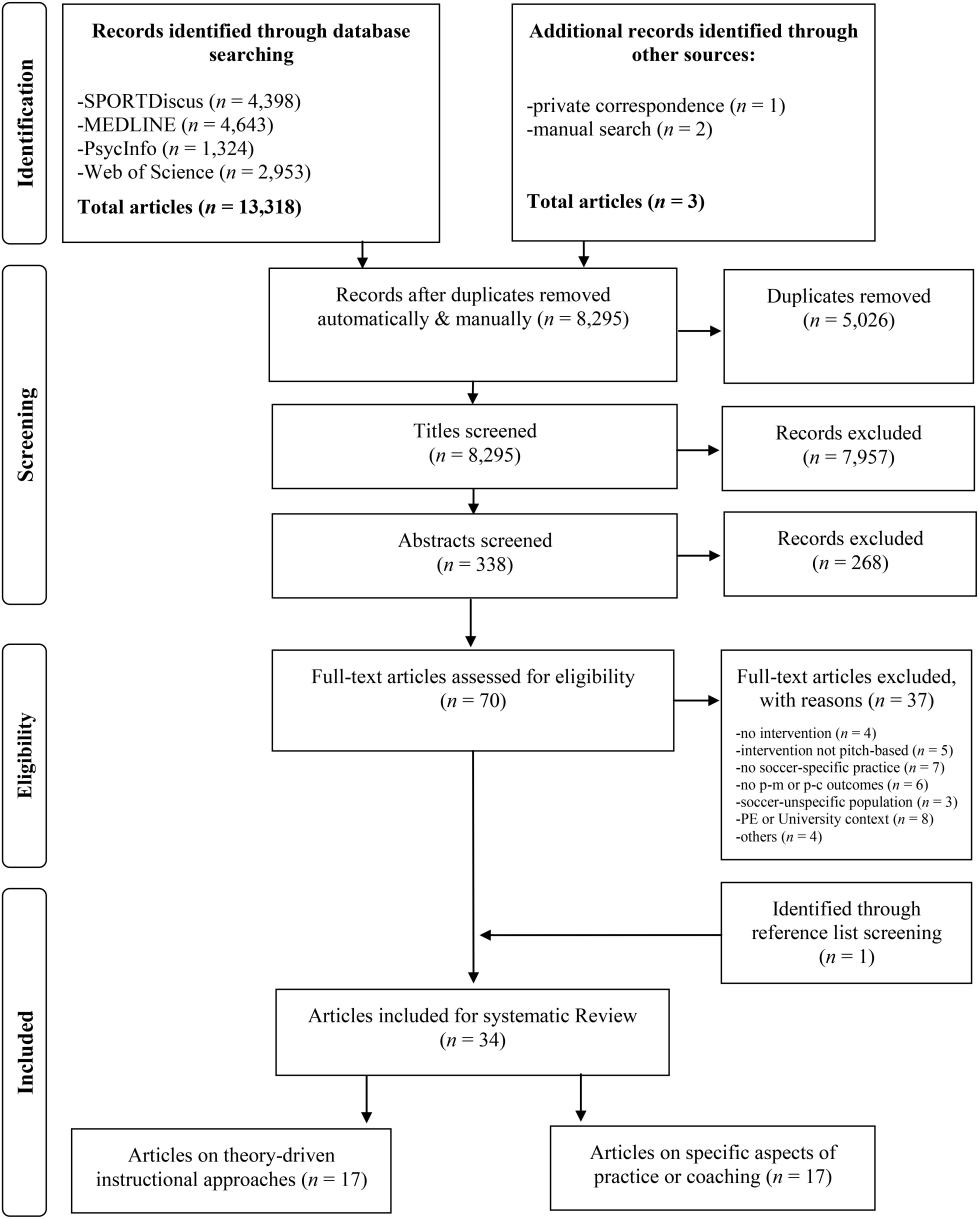
PRISMA flowchart for the documentation of the systematic search process. Notes: The 17 articles on theory-driven instructional approaches include 18 individual studies. PE, Physical Education; p-c, perceptual-cognitive; p-m, perceptual-motor.

The PRISMA flowchart in Figure 1 displays the systematic search results. The initial database search yielded 13,318 hits. Three articles were additionally added through other sources (Hossner et al., 2016; Bozkurt, 2018; Ozuak and Çaglayan, 2019). After removing 5,026 duplicates, 8,295 titles were screened independently by the first author and a trained research assistant. The previously initiated reviewer training comprised an independent title screening of ~10% of identified records and a subsequent discussion of all differently categorized articles based on the inclusion and exclusion criteria. After screening all titles independently, the IRA yielded a sufficient agreement between the two reviewers (97.58%; κ = 0.56). If at least one reviewer argued for inclusion at this stage, the article was moved into the next stage of abstract screening. At this point, 338 potentially relevant abstracts were examined and an excellent IRA was reached (94.67%; κ = 0.92). In case of initial disagreement, study records were discussed by the two reviewers and a decision for or against full-text screening was made. Lastly, 70 full texts were screened independently, the IRA was again found to be excellent (91.43%; κ = 0.87). Uncertainties were discussed with the whole project group. Within this process, 33 articles were identified and one more article was found through manual reference list screening (Schöllhorn et al., 2012). Finally, 34 articles were included for systematic review.

### Data Extraction

The data of included studies were extracted using a Microsoft Excel spreadsheet. All accessible information about the study, participant characteristics, intervention characteristics, instruments, outcome variables, as well as statistical techniques used to assess outcome effects were extracted. To determine whether a quantitative synthesis is possible based on reported data, descriptive and inferential statistics were also extracted. Necessary missing data was requested from the corresponding authors.

### Assessment of Quality in Reporting and Risk of Bias

To judge the quality in reporting and the risk of bias in individual studies, all studies underwent a detailed assessment using an augmented version of the *Downs and Black Checklist* (Downs and Black, 1998) as well as a slightly modified version of the *Template for Intervention Description and Replication* (TIDieR; Hoffmann et al., 2014).^2^

The Downs and Black Checklist (Downs and Black, 1998) was developed for the rating of both randomized and non-randomized studies and has been recently used within systematic reviews and meta-analyses in sports sciences (Davies et al., 2017; Grgic et al., 2018; Ramos et al., 2020). Conventions for the studies' quality scores used in previous reviews were adopted but transferred to percentage as not all items were applicable to all studies. Good methodological quality is represented by a score ≥ 70%. A score ≤ 35% represents a low methodological quality. Scores between these thresholds were considered as moderate.

The TIDieR (Hoffmann et al., 2014) consists of 12 items to assess the quality in reporting of interventions. With the objectives of this systematic review in mind, a detailed intervention description is essential to interpret the results, allow potential replications, and provide coaches with evidence-based recommendations for their work.

To provide a reliable assessment, a similar procedure as for the systematic screening process was applied. An excellent IRA for both the Downs and Black checklist (95.76% [90.9, 100%]; κ = 0.91 [0.71, 1%]) and the TIDieR (96.43% [81.1, 100%]; κ = 0.92 [0.71, 1%]) was achieved. Results for both scales were reported by mean (*M*), standard deviation (*SD*), median (*Mdn*), and range.

### Narrative and Quantitative Synthesis

The study characteristics and results were synthesized narratively for all included studies to provide a descriptive overview of the existent research. Based on this narrative synthesis as well as the judgement of quality in reporting and risk of bias, it was discussed whether an additional quantitative synthesis is possible according to criteria outlined in the PRISMA-P (Bergmann et al., 2020).

Performing a meta-analysis was deemed inappropriate due to the heterogeneity of dependent variables and study designs (e.g., randomized/non-randomized studies; parallel-group/cross-over designs), as well as differences in the studies' quality. More specifically, pooling the effects as done within a meta-analysis was thought to lead to a comparison of dissimilar studies, the inclusion of poorly designed research, and thus, to invalidate results (Tod, 2019; Deeks et al., 2021). However, to estimate the intervention effect sizes in controlled studies, the effect size *d* was calculated in two different ways based on the reported data in the original publications. *First*, if the effectiveness was analyzed by group × time interactions, *d* was calculated from the interaction effect according to Cohen (1988). *Second*, if differences between groups at pre- and post-test were used to analyze the effectiveness (i.e., acquisition effects), *d*_ppc2_ was computed (see Equations 1–3; Morris, 2008; p. 369):


(1) $$\begin{array}{l} d_{ppc2}=c_{P}\;\left[\frac{\left(M_{post,\;T}-M_{pre,\;T}\right)-\left(M_{post,C}-M_{pre,C}\right)}{SD_{pre}}\right] \end{array}$$

where the pooled standard deviation is defined as


(2) $$\begin{array}{l} SD_{pre}=\sqrt{\frac{\left(n_{T}-1\right)SD_{pre,T}^{2}+\left(n_{C}-1\right)SD_{pre,C}^{2}}{n_{T}+n_{C}-2}} \end{array}$$

and


(3) $$\begin{array}{l} c_{P}=1-\;\frac{3}{4\left(n_{T}+n_{C}-2\right)-1} \end{array}$$

The *d*_*ppc*2_ was also applied when multivariate statistics were used to classify effects for single outcome variables. If a retention-test was conducted and all relevant data was available, *d*_*ppc*2_ was calculated using pre- and retention-test data to estimate learning effects.

To consider the intervention duration as a potential moderator, studies were classified as short- (i.e., ≤ 6 sessions or 180 min), mid- (i.e., ≤ 24 sessions or 720 min), and long-term (i.e., ≥ 25 sessions or 2,250 min) interventions. Similar approaches to synthesize intervention effects quantitatively were recently used within systematic reviews in sports science research (e.g., Demetriou and Höner, 2012; Raabe et al., 2019). Results are reported by the range of significant and non-significant effects, the percentage of significant effects, as well as the median when three or more effect sizes were found. Recommendations by Cohen (1988) were used to classify small (*d* ≥ 0.20), medium (*d* ≥ 0.50), and large effects (*d* ≥ 0.80). Effects displayed as positive values represent improvements in the respective outcome in favor of the intervention groups (IGs), while negative values represent greater improvements in control groups (CGs).

## Results

From the 34 identified articles, 85.7% were published between 2010 and 2020, revealing an increase in the number of relevant publications in recent years. The publication by Schöllhorn et al. (2006) includes two studies so that in total 35 individual studies were reviewed. All 35 studies were analyzed regarding their theoretical frameworks and methodological approaches (objectives 1 and 2). To investigate and compare the effectiveness (objective 3), only the 27 controlled studies were considered, while 8 studies without a CG were excluded.

### Theoretical Frameworks and Intervention Content (Perspective 1)

The studies can be grouped into two overarching categories (see Figure 1). The *first group* (*n* = 18) represents studies in which interventions were designed based on theory-driven instructional approaches to practice and coaching. The *second group* (*n* = 17) includes studies investigating specific aspects of either the practice design or coaching behavior and interventions were mostly not grounded in theoretical frameworks.

In the *first group of studies*, interventions were based on mainly three different theoretical and methodological underpinnings, namely DL, TGFU, and NLP (see Table 2). With nine studies, most utilized the DL approach. Of these nine studies, seven compared DL to traditional learning (TL) methods. Additionally, Coutinho et al. (2018) compared an enrichment program of DL to a non-active CG, and Gaspar et al. (2019) investigated acute effects after DL and TL sessions in a single group design.

**Table 2:** Characteristics of studies investigating theory-driven instructional approaches to practice and coaching (*n* = 18).

**Study**	**Country (ISO code)**	**Study design**	**Participants**				**Study description**
			***N* (groups)**	**Age (years)**	**Sex**	**PL**	
**Differential Learning (DL;** ***n*** **=** **9)**							
Bozkurt (2018)	TUR	Pre- to post-test design with CG	15 (2)	Age = 15	n. r.	n. r.	A supplemental drill-based DL program to improve passing, dribbling, and feet-juggling was compared to a drill-based TL program with corrective feedback.
Coutinho et al. (2018)	POR	Pre- to post-test design with CG	30 (4)	*M*_DLU15_ = 14.2 ± 0.8 *M*_DLU17_ = 15.8 ± 0.8 *M*_CGU15_ = 13.9 ± 0.5 *M*_CGU17_ = 16.1 ± 0.7	male	RL	An enrichment DL program to improve attackers' technical skills and creativity was compared to a non-active CG. DL included physical literacy, technical exercises, and SSGs.
Gaspar et al. (2019)	POR	Pre- to post-test design	20 (1)	*M* = 13.8 ± 0.6	n. r.	RL	Acute effects of a session blocked DL in comparison to a session blocked TL with movement feedback. Both sessions aimed at improving goal-shooting velocity and accuracy.
Hossner et al. (2016)	GER	Pre- to post-test design with CG	28 (3)	*M* = 13.8 ± 1.1 Range: 12–15	male	RL	Comparison of drill-based DL without augmented feedback, DL with augmented feedback, and methodologically structured TL to promote players' shooting accuracy.
Ozuak and Çaglayan (2019)	TUR	Pre- to post-test design with CG	52 (2)	*M*_IG_ = 12.03 ± 0.44 M_CG_ = 12.05 ± 0.46 Range: 12–13	n. r.	AL	Drill-based DL, implemented in the regular practice schedule, was compared to a CG that participated in the regular TL practice.
Santos et al. (2018)	POR	Pre- to post-test design with CG	40 (4)	*M*_DLU13_ = 11.1 ± 0.5 *M*_DLU15_ = 13.1 ± 0.3 *M*_CGU13_ = 11.4 ± 0.5 *M*_CGU15_ = 13.0 ± 0.8	n. r.	RL	Game-based DL, focusing on intertrial variability, was compared to game-based practice supported by specific instructions and error correction of a coach. The practice programs are aimed at improving the players' creativity.
Schöllhorn et al. (2006; study 1)	GER	Pre- to post-test design with CG	8 (2)	Adult	n. r.	5^th^ Div.	Drill-based DL to improve passing accuracy was compared to TL based on little inter-trial variability and descriptions of the ideal movement technique.
Schöllhorn et al. (2006; study 2)	GER	Pre-, to post-, and ret-test design with CG	18 (2)	Adult	male	5^th^ & 7^th^ Div.	Blocked DL to improve goal shooting accuracy was compared to TL based on a high number of repetitions and corrective feedback.
Schöllhorn et al. (2012)	GER	Pre-, to post-, and ret-test design with CG	12 (3)	*M*_DLB_ = 24.5 ± 2.1 *M*_DLR_ = 24.5 ± 2.1 *M*_CG_ = 23.8 ± 3.9	n. r.	8th Div.	Random and blocked DL to improve ball control and shooting accuracy was compared to blocked TL focusing on an ideal movement technique and error corrections.
**Teaching Games for Understanding (TGFU;** ***n*** **=** **5)**							
Barquero-Ruiz et al. (2020)	SPA	Pre-test to post-test design	20 (1)^a^	*M* = 9.74 ± 0.79	male and female	LL	The TGFU intervention focused on principles of play in defense and the attack. Each session started with a game form followed by a teaching for understanding period. Technical skills were practiced in drills before returning to a modified game form.
Harvey et al. (2010)	UK	Multiple baseline quasi-experimental design	34 (2)	Range_Firstyear_: 14–15Range_Varsity_: 14–18	male	RC & CP	A TGFU intervention focusing on: “defending as a unit of three players” was conducted. SSGs, phases of play (e.g., offensive vs. defensive on one goal), and functional technical/tactical practice were applied.
Práxedes et al. (2016)	SPA	Quasi-experimental design with CG	18 (2)	*M* = 10.7 ± 0.60	n. r.	YL	TGFU, based on modified games and questioning of the coach to improve the players' offensive tactical behavior, was compared to TL, primarily including technical drills that differed from real game situations.
Práxedes Pizarro et al. (2017)	SPA	Intra-group quasi-experimental design	9 (1)	*M* = 10.55 ± 0.52	Male	RL	A TGFU intervention, including a question-and-answer approach by the coach, to improve the players' decision-making and skill execution was applied. The complexity of the practice program increased progressively during the intervention period.
Sierra-Ríos et al. (2020)	SPA	Non-probabilistic inter-subject case design	30 (2)	*M*_TGFU_ = 10.1 ± 0.10 *M*_DI_ = 10.60 ± 0.57	n. r.	CP	TGFU, based on modified games, was compared to a direct instructional model based on technical and analytical exercises. Interventions aimed at improving players' on- and off-the-ball decision-making and skill execution.
**Non-linear Pedagogy (NLP;** ***n*** **=** **4)**							
Práxedes et al. (2018a)	SPA	Quasi-experimental design	19 (2)	*M*_av_ = 10.55 ± 0.51 *M*_low_ = 10.66 ± 0.50	n. r.	av. & low	Two NLP interventions to develop players' decision-making and skill execution were applied. In the first intervention, SSGs with numerical superiority in attack (+1 player) were conducted. In the second intervention, SSGs with numerical equality were applied.
Práxedes et al. (2018b)	SPA	Quasi-experimental design with CG	19 (2)	*M*_NLP_ = 10.55 ± 0.51 *M*_TL_ = 11.77 ± 0.66	male	LL	The effects of a NLP-intervention, using SSGs with numerical superiority in the attack, were compared to TL, prioritizing technical components. The NLP exercises referred to a principle of play (e.g., maintaining possession of the ball).
Práxedes et al. (2019)	SPA	Intra-group quasi-experimental design	19 (1)	*M* = 10.63 ± 0.49	n. r.	av. to low	Intervention based on the principles of NLP to improve the players' tactical decision-making and skill execution performance. SSCGs with numerical superiority were applied focusing on a tactical principle of play.
Roberts et al. (2020)	UK	Randomized cross-over trial	22 (2)	*M*_IG_ = 16.4 ± 0.4 M_CG_ = 16.1 ± 0.2	n. r.	YA	NLP, based on representative learning designs and perception-action couplings, was compared to a linear information-processing practice program regarding the promotion of attackers' individual learning objectives.

*If authors did not sufficiently report the applied study design, the research group decided on an appropriate descriptive terminology. AL, Amateur Leagues; Av, average; CG, control group; Div., division; DL, differential learning; DLB, differential learning blocked; DLR, differential learning random; IG, intervention Group; LL, local level; NLP, non-linear pedagogy; n. r., not reported; PL, performance level; RL, regional level; TGFU, Teaching Games for Understanding; TL, traditional learning; YA, youth academy; YL, Spanish youth football league*.

a *The 20 participants were randomly divided into two groups that practiced the same content, but the coaches changed between the groups to reduce clustering effects*.

TGFU was investigated in five studies. Two of these compared TGFU with TL, primarily applying technical drill practices supported by direct instructions (Práxedes et al., 2016; Sierra-Ríos et al., 2020). The further TGFU-studies without CGs only investigated within-group changes.

The remaining four studies in this first group investigated NLP. Roberts et al. (2020) compared NLP to promote youth academy attackers' individual learning objectives to a practice program grounded in information-processing theory. Práxedes et al. (2018a) compared NLP to a drill-based and technically focused TL program. Two studies without CG investigated the effects of NLP in a single-group design (Práxedes et al., 2019) or by comparing the effects in different performance groups (Práxedes et al., 2018b).

Within the *second group of studies*, specific aspects of practice and coaching were investigated (see Table 3). Only four studies reported skill acquisition frameworks or discussed the results in the light of theoretical considerations (Weigelt et al., 2000; Haaland and Hoff, 2003; Raastad et al., 2016; Schwab et al., 2019).

**Table 3:** Characteristics of studies investigating specific aspects of practice or coaching (*n* = 17).

**Study**	**Country (ISO code)**	**Study design**	**Participants**				**Study description**
			***N* (groups)**	**Age (years)**	**Sex**	**PL**	
**Effects of technical drill practice (with subsequent SSGs or coordination exercises;** ***n*** **=** **7)**							
Boraczyński et al. (2019)	POL	Single-center, parallel, partially group matched, controlled, and longitudinal design	75 (3)	Range: 10.1–11.9	male	n. r.	Proprioceptive-coordination training (PCT; including 24 technical exercises in combination with coordination exercises) on the players' soccer-specific motor performance was compared to a usual care and a non-active CG.
Holt et al. (2012)	UK	Single subject, multiple baseline experiment	5 (1)	Range: 10–12	male	YA	The effectiveness of the passing-square to promote awareness, passing, and first touch skills was investigated. Based on criteria for successful technical execution, the intervention included individual goal setting, peer-assessed feedback, and group contingency.
Kösal et al. (2020)	TUR	Pre- to post-test design with CG	45 (3)	Range: 10–13	male	n. r.	An additional practice program, including combined technical and coordination exercises to promote soccer-specific technical skills, was compared to regular and unstructured practice CGs.
Miranda et al. (2013)	BRA	Pre- to post-test design	13 (1)	Age = 17	n. r.	NL	The effects of a practice program, including position-specific technical and tactical exercises, as well as gameplay situations on the players' technical performance, were investigated.
Montesano and Mazzeo (2019)	ITA	Pre- to post-design with CG	20 (2)	*M* = 16 ± 0.5	n. r.	CP	The effects of additional technical practice on the players' passing and shooting performance were investigated and compared to a CG without additional practice.
Weigelt et al. (2000)	UK	Pre- to post-test design with CG	20^a^ (2)	Range: 19–20	male	IM	Learning and transfer effects of additional, individual feet-juggling practice without any specific guidance or learning strategies were investigated and compared to a non-active CG.
Zago et al. (2016)^a^	ITA	Pre- to post-test design with CG	20^a^ (2)	*M* = 11.5 ± 0.3	male	RL	Practice, including technical drills and phases of play situations by using tape matrix structures as three-dimensional spatio constraints was compared to a CG that participated in technical drills, SSGs, and situation games without such spatio constraints.
**Practice to reduce lateral asymmetries or improve the non-dominant leg performance (*****n*** **=** **5)**							
Guilherme et al. (2015a)	POR	Randomized cross-over design	50 (2)	*M* = 9.54 ± 1.86	male	EL	The intervention period included additional drill-based practices for improving soccer-specific technical skills in the non-preferred leg. The control period did not include additional exercises.
Guilherme et al. (2015b)	POR	Pre- to post-test design with CG	71 (6)	*M* = 14.44 ± 1.04 Range: 11–16	male	CP	The IG participated in drill-based and technically focused practice with a more frequent use of the non-dominant leg. The CG participated in a practice program by using both legs equally.
Haaland and Hoff (2003)	NOR	Pre- to post-test design with CG	39 (2)	Range: 15–21	male	CP	The IG participated in an increased volume of non-preferred leg practice within the team practice context. The effects were compared to a usual care CG.
Teixeira et al. (2003)	BRA	Pre- to post-test design with CG	24 (2)	Range: 12–14	n. r.	n. r.	The “non-preferred leg group” practiced 45 min in three out of five weekly sessions including drills and SSGs by only using the non-preferred leg. The “preferred-leg group” used both legs equally.
Witkowski et al. (2011)	POL	Pre- to post-test design with CG	37 (3)	Age = 13	male	EL	One group predominantly used the non-dominant leg in technical drills, while another group used both legs equally. The effects were compared to a regular practice CG.
**Effects of game-base practice programs (*****n*** **=** **2)**							
Arslan et al. (2020)	TUR	Experimental parallel matched group design	20 (2)	*M* = 14.2 ± 0.5	male	YA	The effects of game-based practice by using various forms of SSGs were compared to a running-based HIIT training program.
Radziminski et al. (2013)	POL	Pre- to post-test design with CG	20 (2)	*M*_SSG_ = 15.1 ± 0.67 *M*_RG_ = 15.0 ± 0.46	n. r.	n. r.	The effects of a practice program including various forms of 3v3 SSGs on the players' technical actions were investigated and compared to a running-based HIIT program.
**Practice with modified ball sizes (*****n*** **=** **2)**							
Bekris et al. (2012)	GRE	Pre- to post-test design with CG	54 (4)	*M* = 11 ± 0.6	n. r.	n. r.	The effects of technical practice with a size-2 ball in different frequencies and with different content were compared to a CG that practiced with a size-4 ball.
Raastad et al. (2016)	NOR	Pre- to post-test design	17^c^ (2)	*M* = 16.6 ± 0.93 Range: 16–19	male and female	RL	Two groups practiced soccer juggling in two different conditions: One group practiced with a smaller size 1 ball. The other group practiced with a larger size 4 ball. The test ball was a size 3 ball.
**Internal and external focus feedback (*****n*** **=** **1)**							
Schwab et al. (2019)	GER	Pre- to post-, and ret-test design with CG	56 (4)	Adol.: U15-U17 Adults: n. r.	male	LL	The effects of external compared to internal focus feedback on learning the knuckle ball free-kick technique were investigated. Specific instructions were delivered after every third free kick.

*If authors did not sufficiently report the study design, the research group decided on an appropriate descriptive terminology. Adol., adolescents; CG, control group; CP, competitive players; EG, experimental group; EL, elite level; EP = FD, first division; HIIT, High-intensity interval training; IG, intervention group; IM, intermediate Level; LL, Local level; LSPT, Loughborough Soccer Passing Test; NL, national level; n. r., not reported; RG, running group; RL, regional level; SSG, small-sided games; YA, youth academy*.

a *Weigelt et al. (2000): the sample was reduced from 26 to 20 participants as two players did not participate in every session and goalkeepers were excluded from analyses*.

b *Zago et al. (2016): the sample was reduced from 26 to 20 participants that were able to participate in every session and test*.

c *Raastad et al. (2016): the sample was reduced from 22 to 17 participants that completed the practice sessions and were not injured*.

Sixteen studies focused on the design of practice tasks, whereby a multitude of different aspects and outcomes were pursued. For instance, seven studies examined the effects of drill-based practices on technical outcomes. Within these seven studies, two interventions only included deliberate technical drill practices (Weigelt et al., 2000; Montesano and Mazzeo, 2019). Others applied drill-based practices with subsequent game-based situations (e.g., Holt et al., 2012; Miranda et al., 2013) or combined it with coordination exercises (Boraczyński et al., 2019; Kösal et al., 2020).

Another five studies examined technical drill-practice programs focusing on the players' non-dominant leg performance (e.g., Teixeira et al., 2003). Finally, two studies examined game-based interventions (Radziminski et al., 2013; Arslan et al., 2020), and another two the effects of practicing with modified ball sizes (Bekris et al., 2012; Raastad et al., 2016).

Lastly, as the only study on coaches' behavior, Schwab et al. (2019) compared the effects of internal and external focus feedback for learning the knuckle ball freekick technique.

### Methodological Approaches (Perspective 2)

#### Study Designs

Various study designs were used to investigate the influence of practice design and coaching behavior on soccer players' skill acquisition (see Tables 2, 3). Single-group (*n* = 7; 20.0%), as well as multi-groups designs with two (*n* = 19; 54.3%), three (*n* = 6; 17.1%), four (*n* = 2; 5.7%), or six groups (*n* = 1; 2.9%) were found. Across the 27 controlled studies, different practice activities of CGs were found that need to be considered for interpreting intervention effects (see in detail Supplementary Material III). For example, most CGs practiced according to different approaches compared to the IG (*n* = 17) or participated in their regular (“usual care”) practice (*n* = 4). Another three studies compared the interventions to non-active CGs. Only three cases were identified in which different practice or coaching methods as well as usual care or non-active CGs were investigated (e.g., Witkowski et al., 2011).

#### Measurements and Statistical Analyses

In 19 studies (54.3%) two measurement points were assessed (i.e., mostly pre- and post-test). Twelve studies (34.3%) conducted three, and three studies (11.4%) conducted four measurements (e.g., through intermediate measurements). Only six studies (17.1%) conducted a retention test to assess learning effects. Holt et al. (2012) observed the players' performances during the intervention in every session. Five studies that used systematic in-game observations averaged the performances from different matches as values for the respective measurement point (e.g., Práxedes Pizarro et al., 2017).

For assessing outcome effects, most studies used repeated-measures analyses of variance (*n* = 14, 40%). Four studies (11.4%) used multivariate analyses of variance and three studies (8.6%) applied *t*-tests. Another eight studies (22.9%) used non-parametric tests (e.g., *U*-test and Wilcoxon test). Two studies (5.7%) reported the players' improvements descriptively for each player (Holt et al., 2012; Montesano and Mazzeo, 2019) or used non-clinical versions of magnitude-based inferences (Coutinho et al., 2018; Gaspar et al., 2019).

#### Intervention Characteristics

The intervention duration varied substantially across studies (see **Table 5**). Large differences in the intervention duration in weeks (*M* = 11.58, *SD* = 11.63, *Mdn* = 8, [1, 48]), the number of sessions (*M* = 29.41, *SD* = 34.27, *Mdn* = 15, [1, 144]), as well as the sessions' duration in minutes (*M* = 45.00, *SD* = 28.36, *Mdn* = 30, [10, 120]) were found. Further, the number of sessions per week ranged from one to seven sessions (*M* = 2.57, *SD* = 1.24, *Mdn* = 2). Overall, four studies (11.4%) can be classified as short-, 20 studies (57.1%) as mid-, and 10 studies (28.6%) as long-term interventions. One study could not be categorized due to incomplete descriptions (Montesano and Mazzeo, 2019).

#### Participant Characteristics

In total, 992 participants were investigated in the reviewed studies, consisting of on average 28.34 participants per study (*SD* = 17.16, *Mdn* = 20.00, [5, 75]). The number of participants per group ranged from four to 30 (*M* = 13.52, *SD* = 6.36, *Mdn* = 11.66).

Most studies investigated male participants (*n* = 17, 48.6%), two studies (5.7%) examined both males and females (Raastad et al., 2016; Barquero-Ruiz et al., 2020) and 16 studies (45.7%) did not specify participants' sex. Whereas, four studies (11.4%) investigated adult players and two studies (5.7%) examined both youths and adults, most studies utilized youth soccer players (82.9%, *n* = 29). Thereby, most studies were conducted with participants between the age of 11 to 15 (*n* = 20).

Twentynine studies reported the performance level of the sample (82.9%; see Tables 2, 3). A wide range of terminologies was found, in some cases corresponding to national league systems. Regional level players (incl. descriptions such as “moderate level”) were most frequently examined (*n* = 11, 31.4%), followed by studies with youth academy or national level players (*n* = 6, 17.1%) and local (or “low”) level players (*n* = 4; 11.4%). Two studies compared the effects of interventions with players on different performance levels (Harvey et al., 2010; Práxedes et al., 2018b). Studies with adult participants investigated players from regional levels (i.e., 5^th^ to 8^th^ divisions). The youth and adult players in Schwab et al.'s (2019) study were recruited from local levels, Haaland and Hoff (2003) investigated competitive players.

#### Instruments

To assess outcome effects, skill tests were used in 22 studies (62.9%) and systematic observations were applied in 13 studies (37.1%; see in detail Supplementary Material III). No study used both kinds of assessments. The systematic observations occurred in different contexts that included in-game observations during practice (*n* = 7), competition (*n* = 5), or technical drills (*n* = 1). The Game Performance Evaluation Tool (GPET; García López et al., 2013) was most frequently employed for in-game observations (*n* = 6), followed by the Game Performance Assessment Instrument (GPAI; Oslin et al., 1998; *n* = 2), the Creative Behavior Assessment in Team Sports (CBATS; Santos et al., 2017; *n* = 2), and the System of Assessment of Functional Asymmetry of the Lower Limbs in Football (SAFALL-FOOT; Guilherme et al., 2012; *n* = 2). SSGs, ranging from 3v3 to 8v8, were utilized for systematic observations.

#### Quality in Reporting and Risk of Bias

**Figure 2:**
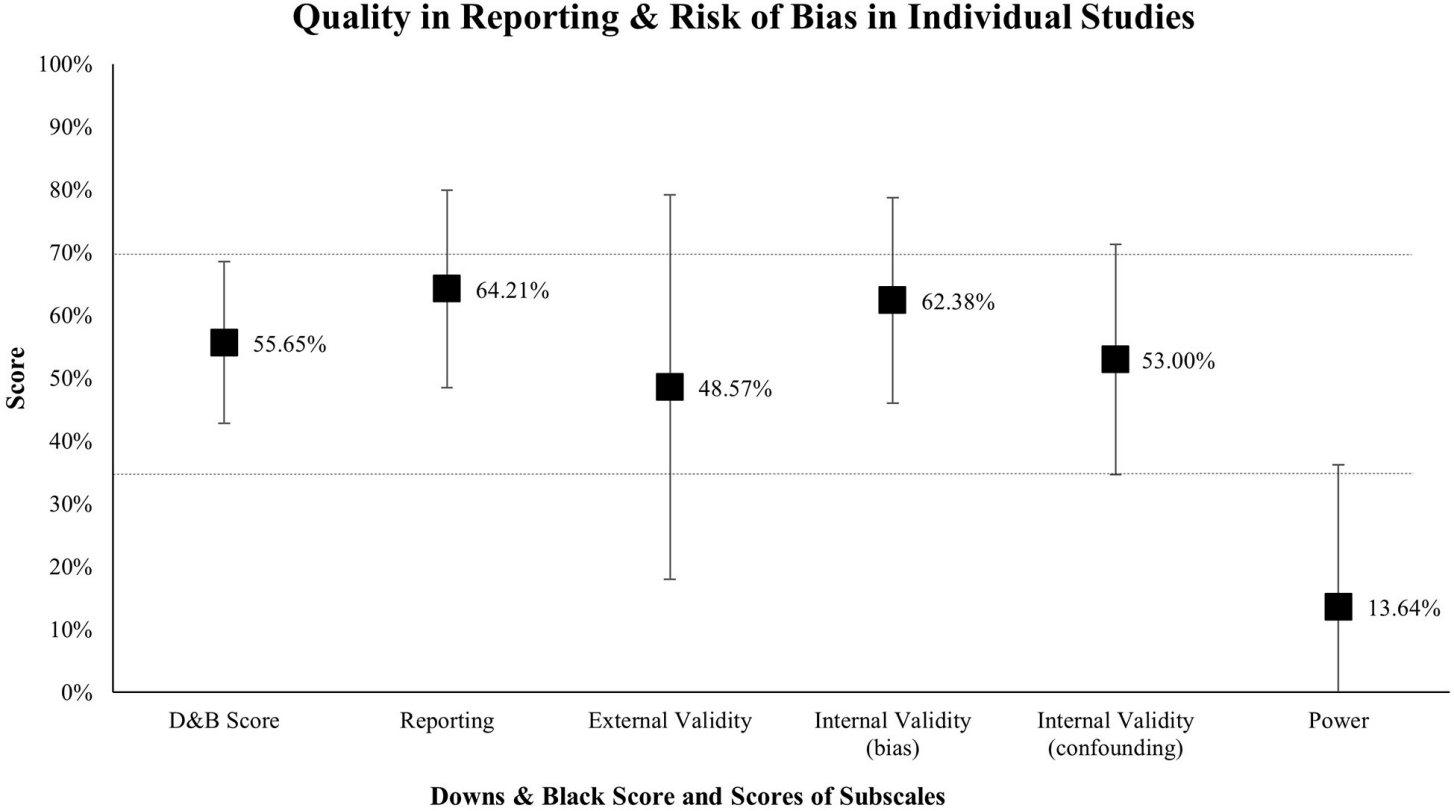
Quality in reporting and risk of bias in individual studies. D&B Score, Downs and Black score (all items). Notes: Results are presented as means with error bars that represent the standard deviation. The dashed lines show thresholds for low (≤ 35%) and high (≥ 70%) scores. It must be considered that the subscales *external validity* and *power* were calculated from only three and two items, respectively.

The results for the assessment of the quality in reporting and risk of bias in individual studies are presented in Figure 2 (see in detail Supplementary Material II). On average, the score of the Downs and Black Checklist reveals a moderate methodological quality (*M* = 55.65%, *SD* = 12.86%, *Mdn* = 57.69%). There are, however, large differences between studies ranging from 30.77% to 78.57% of the total score (2x low score, 5x good score). Most papers lacked a sufficient report of principal confounders (item 5), only four studies (11.4%) met this criterion. To assess outcome effects, 10 studies (28.6%) used assessments that were not evaluated regarding their reliability (item 20). Moreover, very few studies conducted a priori sample size estimations (*n* = 5, 14.3%), leading to an overall low statistical power.

The TIDieR displays large differences across studies with an average score of 59.11% (*SD* = 22.52%, *Mdn* = 66.67%, [11.11, 100]). Even the two studies that reached 100% did not differentiate for every single session but provided an overview of the intervention content across sessions (Zago et al., 2016; Roberts et al., 2020). Another 20 studies (57.1%) presented the procedures in a manner that permits the replication of at least parts of interventions. Lastly, 15 studies (42.9%) controlled as to whether the intervention was delivered as intended, but only nine studies reported underlying criteria of observation.

#### Outcome Variables

A list of examined outcome variables is provided in Table 4. In total, 150 outcome variables were investigated. However, 123 different variants for operationalization (e.g., different skill tests) and different contexts of assessments (e.g., game formats for systematic observations) lead to a highly heterogeneous conglomeration of outcomes. Moreover, six studies employed overall performance scores which included perceptual-motor and perceptual-cognitive characteristics. Yet, none of these scores were calculated based on variables assessed in comparable contexts or with identical equations.

**Table 4:** Soccer-specific outcome variables (*n* = 150) from perceptual-motor and perceptual-cognitive skill domains.

**Perceptual-motor domain (*n* = 105; 85)**	**Perceptual-cognitive domain (*n* = 45; 38)**
*Technical performance (skill tests):*	*Tactical decision-making (in-game):*
Shooting/kicking/striking (*n* = 25; 16)	Decision-making cumulative (*n* = 9; 9)
Dribbling (*n* = 19; 17)	Passing (*n* = 5; 2)
Passing (*n* = 15; 14)	Dribbling (*n* = 3; 2)
Juggling (*n* = 8; 7)	
Ball reception/control (*n* = 7; 6)	*Decision-making (skill test):*
Turns (*n* = 2; 2)	Goal shooting (*n* = 1; 1)
Awareness (*n* = 1; 1)	
Ball bouncing (*n* = 1; 1)	*Defensive tactical behavior (in-game):*
Balance with ball (*n* = 1; 1)	Adjust (*n* = 2; 2)
Heading (*n* = 1; 1)	Covers (*n* = 2; 2)
1v1 (*n* = 1; 1)	
Execution time (LSST; *n* = 1; 1)	*Creativity (in-game):*
	Attempts (*n* = 5; 4)
*Technical skill execution (in-game):*	Fluency (*n* = 5; 4)
Skill execution cumulative (*n* = 8; 8)	Versatility (*n* = 5; 4)
Passes (*n* = 5; 2)	Fails (*n* = 4; 4)
Dribbles (*n* = 4; 3)	Originality (*n* = 4; 4)
(Goal) shots (2; 2)	
Preferred foot performance (*n* = 2; 1)	
Non-preferred foot performance (*n* = 2; 1)	

*Results in brackets represent the n of investigated variables (first number) and the n of different operationalizations (second number). Performance scores that were calculated from both perceptual-motor and perceptual-cognitive skill domains (n = 6) were not displayed in this table. LSST, Loughborough Shooting Skill Test*.

On average, 4.29 (*SD* = 3.39, *Mdn* = 4, [1, 20]) outcome variables were investigated per study. Thirty four studies (97.2%) assessed perceptual-motor outcomes, such as the players' technical performance in skill tests or in-game technical executions. Eleven studies (31.4%) assessed perceptual-cognitive outcomes, such as the divergent (i.e., creativity) or convergent (i.e., decision-making) tactical performance. Two studies examined tactical behavior in defense (Harvey et al., 2010; Barquero-Ruiz et al., 2020), while all other studies addressed aspects in the attack.

Perceptual-motor outcomes, such as shooting accuracy or dribbling were most often collected via skill tests (*n* = 22 studies). Technical skill execution, assessed with the GPET or GPAI, was the most frequently examined in-game variable (*n* = 8 studies). Five studies distinguished between different techniques (i.e., in-game passes or dribbles), while others reported cumulative variables.

Decision-making was the most often examined perceptual-cognitive outcome (*n* = 9 studies). Práxedes et al. (2016, 2018a,b, 2019), Práxedes Pizarro et al. (2017), and Roberts et al. (2020) reported whether the decisions occurred in different contexts (i.e., passing, dribbling, or shooting) while others only provided accumulated performance values. Lastly, in-game creativity was examined in two studies (Coutinho et al., 2018; Santos et al., 2018).

### Effectiveness of Interventions (Perspective 3)

Inferences on the effectiveness of interventions can only be drawn from the 27 controlled studies. The main results were narratively synthesized in Table 5. A summary of results for studies without a CG is provided in Supplementary Material IV. For the quantitative synthesis of controlled studies, 51 effect sizes (26 significant; 50.98%) for perceptual-motor outcomes and 32 effect sizes (11 significant; 34.38%) for perceptual-cognitive outcomes could be recalculated (see Table 6). Given the large differences across studies (e.g., practice activities of CGs) a detailed analysis of individual studies is necessary.

**Table 5:** Narrative synthesis of the effectiveness of interventions in controlled designs (*n* = 27).

**Study**	**Groups**	**Intervention duration**		**Statistical analysis**	**Outcome variables**	**Main results**
		**Sessions (min. p. session)**	**Weeks**			
**Differential Learning (DL;** ***n*** **=** **8)**						
Bozkurt (2018)^a^	- DL (*n* = 6) - TL (*n* = 6)	12 (20)	4	U-Test and Wilcoxon	*Skill tests:* - passing - dribbling - feet-juggling	No significant differences between groups in technical outcomes were found at pre- or post-test. Thus, neither DL nor TL was found to be more effective for promoting soccer-specific techniques.
Coutinho et al. (2018)^a^	- DL U15 (*n* = 9) - DL U17 (*n* = 6) - Usual care U15 (*n* = 9) - Usual care U17 (*n* = 6)	20 (25)	10	Non-clinical magnitude-based inferences	*In-game:* - Technical performance (dribbles, shots, and goals) - Creativity (fluency, attempts, and versatility)	In U15, greater improvements after DL compared to TL in all technical variables, fluency, and versatility were found. In U17, DL only achieved greater improvements in shooting, while no effects in further outcomes were found.
Hossner et al. (2016)	- DL (*n* = 9) - DL and FB (*n* = 9) - TL (*n* = 10)	12 (30)	6	Repeated measures ANOVA	*Skill-test:* - Shooting accuracy	No significant interactions between the DL and TL groups, as well as DL with and without feedback groups, were found.
Ozuak and Çaglayan (2019)	- DL (*n* = 26) - Usual care (*n* = 26)	24 (40–50)	8	U-Test and Wilcoxon	*Skill tests:* - Creative speed test - Dribbling - Juggling - Passing	The DL group achieved significantly greater improvements in creative speed and ball dribbling tests. No significant differences compared to the usual care group were found in juggling and passing.
Santos et al. (2018)	- DL U13 (*n* = 10) - DL U15 (*n* = 10) - TL U13 (*n* = 10) - TL U15 (*n* = 10)	40 (30)	20	ANCOVA	*In-game:* - Creativity (fails, attempts, fluency, versatility, and originality) in passes, dribbles, and shots	DL led to significantly greater effects in few creative components in both ages compared to TL. A decrease in fails in both ages was found. Significant differences were also found in attempts, originality, and most stressed in versatility. More significant and higher effect sizes were found in the U13 age group.
Schöllhorn et al. (2006; study 1)	- DL (*n* = 8) - TL (*n* = 8)	12 (20–40)	4	U-test	*Skill test:* - Passing accuracy with non-dominant foot	Significant differences at post-test between groups reveal a greater effectiveness of DL in non-dominant foot passing accuracy compared to TL.
Schöllhorn et al. (2006; study 2)	- DL (*n* = 8) - TL (*n* = 8)	12 (25)	6	U-test	*Skill test:* - Shooting accuracy	Significantly greater improvements after DL in shooting accuracy were found. DL also outperformed TL after 1-year retention period.
Schöllhorn et al. (2012)	- DL blocked (*n* = 4) - DL random (*n* = 4) - TL (*n* = 4)	8 (25)	4	H-Test	*Skill tests:* - Ball control - Shooting accuracy	In the acquisition phase, only the blocked DL achieved greater improvements in goal shooting. At retention-test only the random DL outperformed TL. No differences in ball control were found.
**Teaching Games for Understanding (TGFU;** ***n*** **=** **2)**						
Práxedes et al. (2016)	- TGFU (*n* = 9) - TL (*n* = 9)	21 (60)	12	MANOVA	*In-game:* - Decision-making (passing and dribbling) - Skill execution (passing and dribbling)	TGFU was found to be significantly more effective than TL in promoting decision-making (passing and dribbling). The only significant difference in the execution variables in favor of TGFU was found for passes.
Sierra-Ríos et al. (2020)^a^	- TGFU (*n* = 15) - DI (*n* = 15)	12 (80)	6	MANCOVA	*In-game:* - Decisions (on- and off-the-ball) - Executions (on- and off-the-ball)	A significantly greater reduction in the number of unsuccessful on-the-ball executions, a decrease in off-the-ball errors, and more successful off-the-ball actions after TGFU were present. No differences in the successful on-the-ball performance were found.
**Non-linear Pedagogy (NLP;** ***n*** **=** **2)**						
Práxedes et al. (2018a)	- NLP (*n* = 10) - TL (*n* = 9)	14 (60)	7	MANOVA	*In-game:* - Decision-making (passing and dribbling) - Skill execution (passing and dribbling)	No significant group × time interaction was found. However, at post-test, the NLP group significantly outperformed the TL group in passing decisions and executions. No differences were found in dribbles.
Roberts et al. (2020)	- NLP (*n* = 11) - IP (*n* = 11)	8 (60)	4	U-Test and Wilcoxon	*Skill-test:* - Strong foot finishing - Weak foot finishing−1v1 - Decision-making	Significantly greater improvements in the NLP group compared to the IP group were found in 1v1 and decision-making skills. No significant differences were found in the technical shooting proficiency or the execution time.
**Effects of technical drill practice (with additional SSGs or coordination training;** ***n*** **=** **5)**						
Boraczyński et al. (2019)^a^	- PCT (*n* = 26) - Usual care (*n* = 27) - Non-active (*n* = 22)	n.r. (30 min. add. practice)	12 months	Repeated measures ANOVA	*Skill tests (dom. leg):* - Turning the ball backward - Slalom dribbling - Static balance with a ball - Kicking accuracy	Only in the static balance test with a ball, a group × time interaction was found due to greater improvements in the PCT group at peri- and post-test compared to the usual care group.
Kösal et al. (2020)	- Coordination (*n* = 15) - Usual care (*n* = 15) - Unstructured (*n* = 15)	30 (30 min. add. practice)	10	Repeated measures ANOVA	*Skill tests:* - Dribbling - Passing - Shooting - Ball bouncing - Wall volley	The coordination group improved in all variables and fewer within-group effects were found compared to the usual care group. The unstructured practice group did not improve in any variable. However, no interaction effects were reported.
Montesano and Mazzeo (2019)	- Add. practice (*n* = 9) - Usual care (*n* = 9)	n. r. (60-80)	n. r.	Descriptive analyses	*Skill tests:* - Passing - Shooting	Both groups descriptively improved in successful passes and goal shots. Descriptively greater improvements were found after add. practice.
Weigelt et al. (2000)	- Intervention (*n* = 10) - Non-active (*n* = 10)	28 (10)	4	MANOVA	*Skill-tests:* - Juggling with feet - Juggling with Knees - Ball control strong foot - Ball control weak foot	A significant time x group effect due to improvements in knee juggling and ball control with both feet (transfer effect), as well as a trend toward better feet-juggling performance, was found.
Zago et al. (2016)^a^	- Intervention (*n* = 10) - Control (*n* = 10)	38 ($\frac{1}{4}$ to $\frac{1}{2}$ of the average session time of 98 min)	22	Repeated measures ANOVA	*Skill tests:* - LSPT - Shuttle dribble test - Slalom dribble test	A significant time × group interaction was found in the LSPT performance (execution time) due to greater improvements in the IG. No significant interactions in other variables were found.
**Practice to reduce lateral asymmetries or improve the non-dominant leg performance (*****n*** **=** **5)**						
Guilherme et al. (2015a)	Cross-over: - Group 1 (*n* = 26) - Group 2 (*n* = 24)	48 (20)	16	Repeated measures ANOVA	*In-game:* - Preferred foot performance utilization - Non-preferred foot performance utilization	The non-dom. leg practice significantly increased the utilization rate during match-play. The interruption of the additional practice during the retention period partially reversed this effect.
Guilherme et al. (2015b)	- NPL U13 (*n* = 12) - NPL U15 (*n* = 11) - NPL U17 (*n* = 12) - CG U13 (*n* = 12) - CG U15 (*n* = 12) - CG U17 (*n* = 12)	108 (20)	36	Repeated measures ANOVA	*In-game:* - Preferred foot performance utilization - Non-preferred foot performance utilization	The experimental practice program led to a significantly greater utilization rate of the non-preferred leg during match-play, while the use of the preferred leg significantly decreased.
Haaland and Hoff (2003)^a^	- Intervention (*n* = 18) - Usual care (*n* = 21)	n. r. (n. r.)	8	Repeated measures ANOVA	*Skill tests:* - Slalom dribbling - Ball control (chest) and volley shooting accuracy - One-touch passing accuracy	Significantly greater improvements in the intervention group compared to the CG in both the dominant and non-dominant legs in dribbling, volley shooting, and one-touch passing variables.
Teixeira et al. (2003)	- 12-year PL (*n* = n. r.) - 13-year PL (*n* = n. r.) - 15-year PL (*n* = n. r.) - 12-year NPL (*n* = n. r.) - 13-year NPL (*n* = n. r.) - 15-year NPL (*n* = n. r.)	80 (45)	16	Repeated measures ANOVA	*Skill tests:* - Speed of dribbling - Kicking for accuracy - Kicking for force	Only in speed dribbling, the lateral asymmetry was significantly reduced from pre- to post-test in the non-preferred-leg group. In other variables, no significant differences between groups were found due to improvements in both the preferred and non-preferred-leg groups.
Witkowski et al. (2011)	- Non-dom-leg (*n* = n. r.) - Both legs (*n* = n. r.) - Usual care (*n* = n. r.)	144 (n. r.)	12 months	t-tests	*Skill tests:* - Dribbling - Ball striking	Both the non-dominant and dominant leg groups achieved greater improvements in technical outcomes compared to the usual care group.
**Effects of game-based practice programs (*****n*** **=** **2)**						
Arslan et al. (2020)	- SSG (*n* = 10) - HIIT (*n* = 10)	10 (10–18)	5	Repeated measures ANOVA	*Skill tests:* - Dribbling speed - Zigzag agility with ball	Both groups improved in their technical performance from pre- to post-test. Higher within-group effects were found in the SSG group. No interaction effects were reported.
Radziminski et al. (2013)^a^	- SSG (*n* = 9) - Running (*n* = 10)	16 (90)	8	Repeated measures ANOVA	*Skill tests:* - Juggling - Rotation pass - Passing - Dribbling - Heading, - Bench passing - Shooting accuracy	No significant group × time interaction was found. The performance increased in both the SSG and Running groups.
**Practice with modified ball sizes (*****n*** **=** **2)**						
Bekris et al. (2012)	- Competitive (*n* = 12) - 20-min (*n* = 13) - 30-min (*n* = 14) - Control (*n* = 15)	12 (20–30)	n. r.	Repeated measures ANOVA	*Skill tests:* - Passing - Juggling - Running with the ball - Turning	Significantly greater improvements in juggling, running with the ball, and turning in all intervention groups compared to the CG were reported. No effects in passing were found.
Raastad et al. (2016)	- Smaller ball (*n* = 11) - Larger ball (*n* = 11)	24 (10)	6	Repeated measures ANOVA	*Skill tests:* - Juggling - Ball reception	The ball juggling performance of both groups increased from pre- to post-test, but no interaction effect regarding transfer effects was found.
**Internal and external focus feedback (*****n*** **=** **1)**						
Schwab et al. (2019)	- Internal adol. (*n* = 10) - Internal adult (*n* = 18) - External adol. (*n* = 10) - External adult (*n* = 18)	6 (20)	3	Repeated measures ANOVA	*Skill test:* - Rotational ball velocity - Linear ball velocity	External focus feedback led to a significantly greater reduction in the rotational ball velocity from pre- to post and pre- to ret-test. No effects on the linear ball velocity were found.

*Add., additional; Adol., adolescent; DI, Direct Instruction; DL, Differential Learning; DL and FB, Differential Learning and Feedback; HIIT, High-Intensity Interval Training; LSST, Loughborough Shooting Skill Test; TL, Traditional Learning; TGFU, Teaching Games for Understanding; NPL, non-preferred leg; PL, preferred leg; SSG, small-sided games*.

a *Further variables, that do not correspond to the perceptual-motor or perceptual-cognitive skill domains (e.g., physiological outcomes), were investigated in the study*.

b *Arslan et al. (2020), Kösal et al. (2020), and Witkowski et al. (2011) did not conduct or sufficiently report interaction effects. Thus, the narrative synthesis is limited to the comparison within group time effects between groups*.

**Table 6:** Recalculated effect sizes for perceptual-motor and perceptual-cognitive outcomes from controlled designs.

**Category (*N* of studies)**	**Study design**		***N*** **of effects**		**Perceptual-motor domain**			**Perceptual-cognitive domain**		
	**UCD**	**CD**	***d***	***d*_**PPC2**_**	***N* of effects (% sig.)**	**Median**	**Range**	***N* of effects (% sig.)**	**Median**	**Range**
DL (9)^a^	1	8	4	43	21 (33.33%)	0.49	−0.15–2.37	26 (23.08%)	0.45	−0.23–1.92
TGFU (5)	3	2	0	10	6 (66.67%)	1.98	0.45–2.80	4 (100%)	1.89	0.90–2.62
NLP (4)^b^	2	2	2	5	5 (20.00%)	0.58	0.17–1.05	2 (50.00%)	0.83	0.60–1.05
Drill-based P. (7)	2	5	6	0	6 (50.00%)	0.73	0.12–1.20	–	–	–
Non-dom. Leg (5)^c^	0	5	10	0	10 (100%)	1.16	0.82–2.91	–	–	–
Game-based P. (2)^d^	0	2	0	0	–	–	–	–	–	–
Mod. ball sizes (2)	0	2	1	0	1 (0.00%)	0.36	–	–	–	–
Instructions (1)	0	1	2	0	2 (50.00%)	0.35	0.11–0.59	–	–	–
**Total (35)**	8	27	25	58	51 (50.98%)	0.74	−0.15–2.91	32 (34.38%)	0.61	−0.23–2.62

*All effect sizes comparing intervention and control groups from pre- to post, but also from pre- to ret-test were included in this overview. CD, controlled designs; UCD, uncontrolled designs. P., Practice, sig., significant. Effect sizes from studies on instructional approaches are provided in the upper half of the table. Effect sizes from studies on specific aspects of practice or coaching are presented below*.

a *Schöllhorn et al. (2012) compared random DL, blocked DL, and TL groups. Only the effects for the comparisons of the random and blocked DL groups compared to the TL group, but not for the comparisons of random and blocked DL groups, were considered*.

b *Práxedes et al. (2018b) used a multivariate analysis of variance including both the perceptual-motor (i.e., skill execution in passing and dribbling) and perceptual-cognitive outcomes (i.e., decision making in passing and dribbling). The non-significant effect (d = 1.05) is once included in the perceptual-motor and perceptual-cognitive domain as no differentiation was possible based on the reported results*.

c *Guilherme et al. (2015a,b) reported the effects of the utilization rate for both the dominant and non-dominant leg. As a reduction of lateral asymmetries was intended, the higher utilization rate of the non-dominant leg, but also the lower utilization rate of the dominant leg is displayed as a positive effect*.

d *No effects for game-based studies could be recalculated due to missing data*.

#### Effectiveness of Instructional Approaches

##### Differential Learning

Comparisons of DL and TL led to ambivalent results across studies (see Table 6). Most studies assessed the effectiveness in the acquisition phase regarding precision-based technical variables (e.g., shooting accuracy). Only three out of thirteen effects reached significance and, thus, reveal greater effectiveness of DL [*Mdn*(*d*) = 0.45; −0.15 ≤ *d* ≤ 2.37]. Regarding time-based tests (i.e., dribbling speed), Ozuak and Çaglayan (2019) found two significant effects when supplemental DL was compared to a usual care CG (0.49 ≤ *d* ≤ 1.22). Hence, Bozkurt (2018) did not confirm the greater effectiveness of DL compared to TL in dribbling (*d* = 0.11). Only two studies applied retention tests to assess learning effects in goal shooting precision [Schöllhorn et al., 2006 (study 1), 2012]. In both studies, significantly greater effects in favor of DL were found, although Schöllhorn et al. (2012) only found a statistically relevant retention-effect for random DL [*Mdn*(*d*) = 1.14; 0.64 ≤ *d* ≤ 1.97; two of three significant].

Coutinho et al. (2018) compared DL, using SSGs and technical drills, to a non-active group. Non-clinical magnitude-based inferences displayed greater acquisition effects for DL in U15 players regarding in-game dribbles and shots, as well as creative components fluency and versatility. In U17, greater effectiveness was only found in the technical shooting performance. Santos et al. (2018) found positive effects in favor of game-based DL compared to a game-based CG in few creative components. More significant and slightly higher effects in the U13 [*Mdn*(*d*) = 0.40; 0 ≤ *d* ≤ 1.08; four of 15 significant] compared to U15 [*Mdn*(*d*) = 0.27; −0.08 ≤ *d* ≤ 1.91; two of 15 significant] were found. The largest and most frequent effects were present for versatility in passes and dribbles [*Mdn*(*d*) = 1.05; −0.02 ≤ *d* ≤ 1.92; three of four significant]. Further, DL significantly outperformed TL regarding originality in passes in both ages (0.45 ≤ *d* ≤ 0.68) and fewer fails in dribbles in U13 (*d* = 0.87; Santos et al., 2018).

To sum up, two studies provide support on the general effectiveness of DL in at least a few technical or tactical outcomes (Coutinho et al., 2018; Ozuak and Çaglayan, 2019). Results on the relative effectiveness of DL compared to TL regarding the promotion of soccer-specific techniques are, however, ambivalent. Yet, no study found significantly greater improvements after TL. In terms of creativity, Santos et al. (2018) indicate the potential of DL to encourage versatility and originality compared to other game-based models.

##### Teaching Games for Understanding

The TGFU approach was compared to TL, primarily including decontextualized drills (Práxedes et al., 2016; Sierra-Ríos et al., 2020). Práxedes et al. (2016) found strong effects in favor of TGFU in decision-making for passes and dribbles (0.90 ≤ *d* ≤ 1.40; two of two significant). A significant effect in the execution variables was present for passes (*d* = 1.10), but not for dribbles (*d* = 0.45). In the study by Sierra-Ríos et al. (2020), TGFU outperformed the direct instruction group regarding significantly more successful off-the-ball decisions, and executions (2.62 ≤ *d* ≤ 2.80; 2 of 2 significant), as well as less unsuccessful actions (2.37 ≤ *d* ≤ 2.48; 2 of 2 significant). Regarding on-the-ball variables, only significantly fewer inefficient technical actions support the greater effectiveness of TGFU (*d* = 2.48), but not more efficient technical executions were found.

##### Non-linear Pedagogy

Práxedes et al. (2018a) compared NLP to TL group that prioritized technical components. NLP was altogether not significantly more effective than TL (*d* = 1.05).^3^ However, at post-test, the NLP group significantly outperformed the TL group in passing decisions and executions. No significant differences in dribble variables were found. Roberts et al. (2020) compared NLP to a practice program based on information-processing theory and found greater improvements after NLP in 1v1 (*d* = 0.74) and decision-making skills (*d* = 0.60). No significant differences occurred in technical variables [*Mdn*(*d*) = 0.44; 0.17 ≤ *d* ≤ 0.58].

#### Effectiveness of Interventions on Specific Aspects of Practice or Coaching

Generally, the low number of effect sizes within this second group, as well as an often limited content-related comparability of studies impede an accumulated report. For game-based interventions, even no effect size could be recalculated.

Sixteen controlled studies investigated multiple aspects of the practice design to promote technical skills. Regarding *technical drill practices (with subsequent SSGs)*, Zago et al. (2016) found that the use of tape-matrix structures as spatio temporal constraints for both, technical drills, and phases of play situations, led to a significantly faster passing execution time compared to a group that practiced without such constraints (*d* = 1.05). No interactions in other precision- or time-based technical outcomes occurred [*Mdn*(*d*) = 0.14; 0.12 ≤ *d* ≤ 0.94]. Weigelt et al. (2000) found a significant interaction of deliberate juggling practice compared to a non-active CG, resulting from improvements in knee-juggling, but also positive transfer effects to ball control performance (*d* = 1.20). Such positive transfer to ball control was not confirmed by Raastad et al. (2016), additionally, no differences occurred in deliberate juggling practice with smaller or larger balls (*d* = 0.36).

The *non-dominant leg practice* interventions consistently led to greater effects compared to CGs. Guilherme et al. (2015a,b) found that additional drill-based non-dominant leg practice led to a higher utilization rate in game-based situations compared to non-additional practice CGs (2.31 ≤ *d* ≤ 2.91; two of two significant). The decrease in the preferred leg utilization rate reveals fewer lateral asymmetries during match-play (1.40 ≤ *d* ≤ 2.31; two of two significant). Haaland and Hoff (2003) further showed that the predominant use of the non-dominant leg within team practice led to improvements in both the non-dominant [*Mdn*(*d*) = 0.93; 0.88 ≤ *d* ≤ 1.32; three of three significant] and dominant legs [*Mdn*(*d*) = 0.95; 0.82 ≤ *d* ≤ 0.99; three of three significant] compared to a usual care CG.

In the only study on *coaches' instructions* (Schwab et al., 2019), external focus feedback led to greater improvements compared to internal focus feedback in the knuckle-ball technique due to a reduction in the rotational ball velocity (*d* = 0.59). No differences were found in the linear ball velocity (*d* = 0.11).

## Discussion

Enhancing soccer players' skill acquisition is an essential part of coaches' work that should be supported by evidence-based knowledge to identify the most effective practice and coaching methods. Thirty-five studies were reviewed to pool the growing body of research investigating perceptual-motor and perceptual-cognitive skills as outcomes of interventions anchored in pitch-based settings. Two groups of studies were identified within the present research. In the first group, theory-driven instructional approaches were investigated and compared to non-active CGs or active controls practicing according to differing methodologies. In the second group, specific aspects of the practice design or coaches' instructions were examined, but interventions were often not explicitly embedded within skill acquisition paradigms. In both groups, methodological differences in terms of the study designs, practice activities of CGs, outcome variables, as well as research quality challenge the comparability of the respective study results. Thus, interpreting the potential of the investigated methodologies requires a detailed discussion of underlying theoretical frameworks, derived principles to practice and coaching, as well as studies' methodological characteristics and limitations.

### Theoretical Perspectives

Regarding studies on instructional approaches, underlying frameworks of self-organization approaches DL (i.e., dynamical systems theory) and NLP (i.e., ecological dynamics), as well as corresponding principles for practice and coaching, were clearly stated. Allocating TGFU solely based on skill acquisition theory seems inappropriate as it emerged from “an educative perspective rather than approaching it from purely the field of sports science/skill acquisition” (Harvey et al., 2018; p. 175). In contrast to the underpinnings of IGs' practice, theoretical frameworks of CGs were only stated in a few studies (e.g., Schöllhorn et al., 2006; Roberts et al., 2020). In many studies on TGFU and NLP, the respective CGs' practice was not explicitly linked to a cognitive, information-processing approach to skill acquisition. Instead, CGs were defined on a practical rather than on a theoretical level by “prioritizing the technical component” (Práxedes Pizarro et al., 2017, p. 187) while IGs practiced both technical and tactical aspects of play. Similarly, not withstanding the outlined exceptions, the vast number of studies on specific aspects did not design their interventions based on skill acquisition theory. Thus, many of the reviewed studies only contribute to a practical discussion and do not allow inferences on the explanatory value of skill acquisition theories, and thereto derived methodological conclusions on how to effectively design and deliver practice and coaching.

### Practical Implementation

Typically, technical practices following an information-processing viewpoint targeted the development of “ideal movement archetypes” (Schöllhorn et al., 2012, p. 104) mostly through decontextualized drills. When aspiring to tactical objectives, practices were sometimes enriched by subsequent game-based situations to apply the to-be-learned skills in game-realistic contexts. Coaches acted as “expert[s] leading participants to a series of pre-determined outcomes” (Roberts et al., 2020, p. 1456) by using a high number of verbal instructions and demonstrations. However, it was often not clearly outlined if, when, and how the decomposed technical practice was linked to tactical aspects within the sessions' microstructure and the interventions' periodization.

Regarding DL, a variety of implementations regarding both technically focused drill-based and game-based interventions were utilized. For instance, “superfluous exercises” (Schöllhorn et al., 2006, p. 191) were added to target drills (e.g., additional body movements) or random modifications of SSGs (e.g., equipment or rules) were applied to create noise throughout the learning process. Nevertheless, a hitherto unresolved problem is the lack of metrics for quantifying variability (“noise”) limiting opportunities for replications and practical recommendations for coaches' work.

Both NLP and TGFU interventions primarily include game-based activities and in some cases applied skill practice tasks aiming at a rather natural variability within and between skills. This was mostly achieved through simplified “contexts of play” (Práxedes et al., 2019, p. 335) such as numerical superiority SSGs. These methodological similarities are in line with those outlined in the literature on TGFU and the CLA that is underpinned by principles of NLP (Renshaw et al., 2016; Harvey et al., 2018). Nevertheless, specific differences grounded in the respective theoretical underpinnings were often difficult to identify. Few studies reported in detail how coaches guided players in TGFU in an explicit process through reflective questions (Barquero-Ruiz et al., 2020). Otherwise, more implicit strategies in manipulating constraints to promote “strong functional couplings of information and movement” (Roberts et al., 2020, p. 1456) were described for NLP interventions. Yet, the implementation of specific strategies within a defined context, the triggers for their use, as well as the dose were scarcely specified. Such details in the light of the targeted outcomes are, however, particularly important to achieve conceptual clarity and reduce misinterpretations (Cope and Cushion, 2020).

### Critical Appraisal of Methodological Study Characteristics

The present review faces often outlined methodological challenges in systematic reviews and meta-analyses of intervention research, such as large methodological diversity across studies, outcome variables, and practice activities of CGs (Abad Robles et al., 2020; Silva et al., 2021; Tassignon et al., 2021). Besides that, the quality in reporting and risk of bias, as well as the quality of intervention description largely differed across studies and some general limitations in the present body of research were uncovered. These limitations need to be critically discussed to ensure a careful and reflected interpretation of the interventions' effectiveness.

#### Study Designs and Intervention Characteristics

Studies without a CG need to be necessarily excluded from analyses on the effectiveness as the outcomes could be biased by various confounding factors. Even within controlled studies, the research designs varied substantially, limiting the comparability of results. Caution is also required when comparing the effects as different types of CGs were identified (see in detail Supplementary Material III). While comparisons to non-active CGs only allow conclusions on the general effectiveness of interventions, usual-care CGs provide evidence on whether interventions could improve current practice (Smelt et al., 2010). Attention is advised when CGs mainly practice technical aspects while IGs focus on both technical and tactical content, in particular when both technical and tactical outcomes were assessed through systematic in-game observations. Such comparisons neither allow a theory-led discussion of results, nor enable conclusions on the effectiveness of investigated approaches in relation to other methodologies.

The only studies which allow for conclusions on the relative effectiveness of practice and coaching methods are those that explicitly state theoretical and methodological principles of CGs and by approaching similar practice objectives. However, most studies did not include additional non-active CGs so that improvements in both groups could not be interpreted (e.g., Hossner et al., 2016). Yet, ensuring both sufficient statistical power and including additional no-training CGs could be particularly difficult in applied experimental settings. Nevertheless, in line with Vater et al. (2021), including at least a non-active CG can be seen as the minimum requirement to ensure sufficient quality of evidence. Potentially, randomized crossover designs—as chosen by Roberts et al. (2020)—provide solutions that every player can profit from interventions by simultaneously controlling intervention effects.

Primarily, studies focused on performance changes (i.e., temporary fluctuations in behavior), but not on learning effects (i.e., permanent changes over time; Soderstrom and Bjork, 2015). Such short-time effects may be of great importance within high-performance settings when the goal is to quickly achieve a specific outcome. From a talent development viewpoint, however, developing players' skills systematically would benefit from knowledge on learning effects, thus, requiring the inclusion of retention tests within intervention designs. Additionally, knowledge about methods resulting in positive transfer to other skills but also from decontextualized to game-realistic settings would provide valuable information on how to effectively prepare players for the dynamic nature of the game.

A further identified concern relates to the scarce description of interventions which limits the potential to replicate the reviewed studies. Besides that, little consideration was dedicated to the intervention adherence and fidelity. Systematic assessments based on specific and outlined criteria of observation provide transparent insights on the intervention delivery (e.g., Práxedes et al., 2019). Especially within the highly dynamic and partly unpredictable pitch-based work, systematic assessments would add valuable information on the interventions' protocol adherence.

#### Outcome Variables and Statistical Analysis

The multitude of dependent variables illustrates the variety of relevant outcomes even within the perceptual-motor and perceptual-cognitive skill domains. Yet, multiple ways for measuring the same skills underline that there is no consensus regarding standard assessment tools (Williams et al., 2020). Additionally, instruments lacking a scientifically sound investigation of reliability and validity were found. Those results need to be interpreted with caution. Besides outcome-related skill tests, also an increasing number of in-game assessments were applied. These provide higher ecological validity and the opportunity to assess the functional application of practiced skills (for a similar discussion see Koopmann et al., 2020). However, many interfering factors (e.g., performance of other players) need to be controlled. Consequently, combining skill tests and in-game assessments is recommended.

A further prevalent issue was the low statistical power due to small sample sizes. Unsurprisingly given this result—and in line with similar investigations in sports science research (Abt et al., 2020)—only five studies reported a priori sample size estimations. Further, correlations among repeated measures in individual studies were unknown and may have resulted in less precise *post-hoc* power calculations. Besides inferential statistics, two studies used magnitude-based inferences, however, it is intensively discussed within sports science whether the use of such parameters is adequate as they are often used to justify small sample sizes (Lohse et al., 2020). Additionally, insufficient reports or interpretations of statistical results, missing interaction effects, or unknown group differences challenged the recalculation and interpretation of effect sizes.

Overall, these limitations reinforced the decision to refrain from a meta-analysis and need to be recognized when interpreting and comparing the effectiveness of interventions. Due to the diverse approaches, many studies must be considered in the light of individual characteristics.

### Effectiveness of DL, TGFU, and NLP Interventions

A fundamental question arising from theoretical camps is as to whether practice should aim to improve ideal “textbook” techniques or allow exploration for individual solutions. Seven studies on DL dealt with this question, but large methodological differences and a low statistical power must be considered.

Only a few significant effects were found that confirm the superiority of DL compared to TL methods. Nevertheless, no study reported significantly greater effectiveness of TL methods, permitting the conclusion that DL seems to be at least a viable alternative for practicing technical soccer skills. Supporting this notion, comparisons to non-active CGs provide evidence that DL could generally improve performance (e.g., Ozuak and Çaglayan, 2019). Yet, besides two exceptions, the performance was assessed within skill tests. Thus, investigations that DL supports “more effective and more stable movement patterns” (Schöllhorn et al., 2012, p. 102) within match-play have been widely neglected so far. Further, the greater effectiveness of DL in the retention phase compared to the acquisition phase as found in a meta-analysis by Tassignon et al. (2021) could not be proofed due to the absence of retention tests in most studies.

The two studies on game-based DL support its potential to promote divergent tactical behavior in early- to mid-adolescent regional level players (e.g., Santos et al., 2018). Although generalizable conclusions seem premature due to only two game-based studies on divergent tactical outcomes, the results provide support for a developmental framework on promoting creativity in team sports, recommending DL as one appropriate method during adolescence (Santos et al., 2016).

In contrast to DL, studies on TGFU and NLP consistently aimed at promoting technical and convergent tactical skills (i.e., decision-making). Generally, positive effects regarding the promotion of perceptual-motor and perceptual-cognitive skills indicate the potential of both approaches although only two controlled designs were found. Besides, many studies rather provide evidence on the general effectiveness as targeted practice objectives in IGs (technical and tactical content) and CGs (primarily technical content) seem hardly comparable.

The predominant use of SSGs with numerical superiority led to greater effects in passing variables (e.g., Práxedes et al., 2018a). Consequently, if improvements in dribbling should be targeted, other strategies to simplify game demands are required. As a general result, more significant effects in perceptual-cognitive compared to perceptual-motor variables were found, probably due to the predominant focus on technical skills in CGs. Further, intervention periods longer than eight sessions as used by Roberts et al. (2020) or greater consideration of the technical elements (e.g., applied technical practice) may achieve greater effectiveness in the perceptual-motor domain.

### Effectiveness of Interventions Focusing on Specific Aspects of Practice or Coaching

Providing a clear image of the effectiveness of studies on specific aspects to practice and coaching is challenged as diverse topics within skill acquisition research, practice methods, and technical outcomes were found. Further, most technical outcomes were only investigated through skills tests limiting the inferences which can be drawn for match-play performances. Nevertheless, most studies support the effectiveness of deliberate technical practice although specific study objectives and particularities of applied methods often require individual analyses. Specifically, positive results were reported after technically focused drill-based interventions to promote the technical non-dominant leg performance. Thus, interventions that primarily include TL methods—often applied as supplemental to regular team practice—were found to achieve improvements. Again, none of these studies assessed its impact on match-play performance. Only two studies provide support that drill-based practices over several months can positively impact match-play behavior in terms of a higher utilization rate of the non-dominant leg (Guilherme et al., 2015a,b). In summary, the present research on specific aspects provides first insights on the potential of different practice and coaching methods, but evidence on whether such practices can improve match-play performance is rare.

### Limitations of This Systematic Review

The limitations of the present review mainly relate to three characteristics. *First*, due to large heterogeneity and methodological weaknesses across studies, pooling effect sizes within a meta-analysis to increase the statistical power and precision of effects was not possible. Besides the limited number of controlled designs on comparable approaches, not all effect sizes could be recalculated due to missing data, as well as unknown interaction effects or group differences. Consequently, inferences on the effectiveness of interventions were limited, they could not be statistically investigated regarding potential moderators (e.g., the intervention duration) and, thus, must be often interpreted in the light of individual study characteristics. Along with this, the extent of potential publication biases could not be statistically assessed. *Second*, strict inclusion criteria were applied to ensure the highest quality of individual studies (e.g., peer-reviewed research). Nevertheless, relevant studies published within different outlets (e.g., book chapters) may have been omitted as a consequence. *Third*, improvements in players' perceptual-motor and perceptual-cognitive skills were chosen from a wide range of potentially relevant outcomes (Nichol et al., 2019). Thus, skill acquisition needs to be considered at the interface of various interrelating elements, such as motivational or physiological attributes.

### Future Perspectives

The growing number of studies over the past years elucidated the increasing interest and relevance of the topic. To encourage and improve further work, directions and recommendations for future studies are outlined relating to three main features.

First, *higher standards of intervention research* should be applied, especially in terms of sufficiently powered and controlled designs. Although guidelines for intervention research from health science or medicine may need to be translated and adapted for sports coaching research (e.g., O'Cathain et al., 2019), they can be valuable benchmarks for future work. Further, scientifically grounded and clearly outlined hypotheses are required to better understand what interventions aim to achieve. Pre-registrations could improve the current practice by outlining the study design, hypotheses, statistical analyses, and a priori sample size estimation.

Second, the specific practice objectives of different practice and coaching methods should be critically juxtaposed to conduct *theory-driven intervention research on the relative effectiveness* of approaches (for an example see Gray, 2020). Here, the additional use of non-active or usual care CGs would allow interpreting improvements in different practice groups. The application of instruments with high specificity is recommended to draw sound conclusions on the respective intervention objectives. These can be both outcome-related skill tests, but also representative in-game assessments if potential confounders could be controlled. Additionally to hypothesis testing based on group means, looking at individual development curves would allow specific implications on how different methods impact acquisition and learning in the light of personal characteristics (Anderson et al., 2021).

Third, systematic *monitoring of practice and coaching* would help to better understand and compare the implementation of different approaches. This requires empirically grounded metrics to quantify practice (e.g., variability) and coaching (e.g., instructions). Although proper metrics are scarce, systematic observation instruments can provide valuable data (e.g., Cushion et al., 2012b). Potentially relevant variables may also be adapted from periodization frameworks on skill acquisition (Farrow and Robertson, 2017; Otte et al., 2019). Collaborations with coaches who transfer methodological principles to their “real-world” coaching contexts could show how differently grounded approaches can be applied in different settings. This could specifically profit from mixed-method designs focusing on both the players' outcomes, but also coaches' intentions, expectations, and experiences when applying different methodologies.

### Practical Implications

The present findings underline the need for coaches to make numerous considerations before deciding for or against a specific practice and coaching methodology. These may include, for example, coaches' reflections on which competencies they aim to improve in their players (i.e., the practice objectives) as well as the situative circumstances in which practice is conducted (e.g., age groups or sport facilities). Within coaches' daily work, but beyond the scope of the present review, these considerations cannot be limited to perceptual-motor and perceptual-cognitive skills, but need to go further (see, for example, Alves et al., 2017 for reasons on the use of SSGs in soccer practice).

Regarding the promotion of outcomes addressing *technical skills*, the available results support that both decomposed, repetition-based approaches as well as self-organziation/variability-based approaches implemented within drills or games can lead to improvements depending on how performance is operationalized. These findings challenge the traditional idea that players must learn the “fundamentals” first (e.g., ball handling) before they can be put into the game context (Newell, 2020). Given the other benefits of self-organization/game-based practice activities (e.g., more opportunities for decision-making), this suggests that, at very least, coaches should reduce the amount of decomposed, isolated drills in practice. Here, the key challenge is to find the most supportive integration and periodization of such practices, as well as the optimal degree of self-exploration and variability by considering individuals' requirements and needs.

Regarding *convergent tactical skills* (i.e., decision-making), the present results allow the cautious conclusion that those approaches which provide players a greater opportunity to self-explore tactical solutions within game-realistic settings provide a better foundation to facilitate convergent tactical thinking. Knowledge of how and to what degree this process can be most effectively guided through implicit or explicit coaching strategies is still scarce as comparisons of specific strategies are lacking. In terms of players' *divergent tactical behavior*, it seems worthwhile to confront players with highly dynamic and unpredictable match-play situations that encourage flexible adaptions to game demands. Nevertheless, specific recommendations on the most conducive degree of variability and unpredictability cannot be made based on the present knowledge.

## Conclusion

The small number of studies investigating similar approaches, heterogeneity across studies and dependent variables, as well as methodological weaknesses, limit the generalizability of results. Although it was possible to outline the potential of different practice and coaching methods regarding a variety of outcomes, most effects need to be critically interpreted in the light of individual study characteristics and weaknesses. Thus, based on the current body of knowledge, drawing scientifically sound conclusions on the effectiveness or even superiority of specific approaches would be premature. Rather, the present review aims to encourage further theory-driven and high-quality studies to extend the growing but still limited body of research conducted in applied experimental contexts. Furthermore, the findings must be systematically enriched by coaches' experiential knowledge. This will contribute to a conscious and evidence-based use of the coaches' methodological “toolbox” for effectively enhancing player learning and performance.

## Data Availability Statement

Further data, as well as the PRISMA checklist, are provided in Supplementary Materials I-V. Datasets of the systematic search and the quantitative synthesis can be accessed in the OSF project of this publication (https://osf.io/85tjg/).

## Author Contributions

FB, RG, SW, and OH: conceptualization, review, and editing original draft. FB: methodology, investigation and formal analysis, and visualization and writing original draft. OH: project administration and supervision, and funding acquisition. All authors contributed to the article and approved the submitted version.

## Funding

This study was part of the research project “Scientific Support of the DFB's Talent Promotion Program”, which was granted by the German Football Association (Deutscher Fußball-Bund, DFB). The funder was not involved in the study design, collection, analysis, interpretation of data, the writing of this article or the decision to submit it for publication. We also acknowledge support by Open Access Publishing Fund of the Eberhard Karls University Tübingen. This funding supported the payment of the publication fee.

## Conflict of Interest

The authors declare that the research was conducted in the absence of any commercial or financial relationships that could be construed as a potential conflict of interest.

## Publisher's Note

All claims expressed in this article are solely those of the authors and do not necessarily represent those of their affiliated organizations, or those of the publisher, the editors and the reviewers. Any product that may be evaluated in this article, or claim that may be made by its manufacturer, is not guaranteed or endorsed by the publisher.

## Footnotes

^1^For further explanations see for example: https://www.youtube.com/watch?v=cCsezh7ijzs.

^2^The modified checklists, including further specifications of the items, are accessible in the OSF project for this publication (https://osf.io/85tjg/).

^3^Práxedes et al. (2018b) used multivariate statistics and did not report baseline statistics so that only the MANOVA effect, considering both perceptual-motor and perceptual-cognitive outcomes, can be provided.
