# Supplementary material for: Perceptual-Motor and Perceptual-Cognitive Skill Acquisition in Soccer: A Systematic Review on the Influence of Practice Design and Coaching Behavior
Source: Front Psychol. 2021 Dec 2;12:772201. doi: 10.3389/fpsyg.2021.772201 (PMC8675907; doi:10.3389/fpsyg.2021.772201)
Supplement: Supplementary file 1 [file Data_Sheet_1.pdf]

Supplementary Material

Table of Contents

Supplement I: Systematic Search Documentation and Database Settings.. 2

Supplement II: Quality in Reporting and Risk of Bias in Individual Studies 4

Supplement III: Detailed Description of Intervention Content and Methods 7

Supplement IV: Narrative Summary of Results from Studies without a Control Group 18

Supplement V: PRISMA Checklist 20

Supplement I: Systematic Search Documentation and Database Settings

Documentation of the systematic database search (November 22nd, 2020) of each of the four electronic databases: SPORTDiscus, MEDLINE, PsycInfo & Web of Science (including the categories Sports Science & Psychology).

For each of the searches listed below, the databases were searched from their inception or date of the earliest available publication. The reported times in brackets for the coverage represent the end of the search in the respective database.

**SPORTDiscus**<br>**Platform:** EBSCOHost	**Coverage:** 1965 – 22.11.2020 (11:54 CET)<br>**Hits:** 4,408
SPORTDiscus was searched with the following filters: peer-reviewed, academic journal & English language.<br><br>**Search Strategy:**<br>(football* OR soccer) [Title & Keywords]<br>AND<br>(intervention OR train* OR program* OR approach OR pract* OR effect* OR impact OR improv* OR learn* OR perform* OR coach* OR "skill acquisition" OR cognit* OR ecologic* OR con-straints OR "information processing") [Title & Keywords]<br>NOT<br>(novice OR referee OR injur* OR pupil* OR class OR goalkeep* OR NFL OR "american football" OR “australian football") [Title & Keywords]	
**MEDLINE**<br>**Platform:** EBSCOHost	**Coverage:** 1965 – 22.11.2020 (12:12 CET)<br>**Hits:** 4,643
**Note:** MEDLINE was searched for titles and “MW Word in Subject Heading”, which is equivalent to the “Keyword” search in the other databases.<br><br>**Search Strategy:**<br>(football* OR soccer) [Title & MW Word in Subject Heading]<br>AND<br>(intervention OR train* OR program* OR approach OR pract* OR effect* OR impact OR improv* OR learn* OR perform* OR coach* OR "skill acquisition" OR cognit* OR ecologic* OR con-straints OR "information processing") [Title & MW Word in Subject Heading]<br>NOT<br>(novice OR referee OR injur* OR pupil* OR class OR goalkeep* OR NFL OR "american football" OR “australian football") [Title & MW Word in Subject Heading]	

<table>
<tr><td>PsycInfo
Platform: EBSCOHost</td><td>Coverage: 1914 – 22.11.2020 (12:23 CET)
Hits: 1,324</td></tr>
<tr><td colspan="2">PsycInfo was searched with the following filters: Peer Reviewed & English language

Search Strategy:
(football* OR soccer) [Title & Keywords]
AND
(intervention OR train* OR program* OR approach OR pract* OR effect* OR impact OR improv* OR learn* OR perform* OR coach* OR "skill acquisition" OR cognit* OR ecologic* OR constraints OR "information processing") [Title & Keywords]
NOT
(novice OR referee OR injur* OR pupil* OR class OR goalkeep* OR NFL OR "american football" OR “australian football") [Title & Keywords]</td></tr>
<tr><td colspan="2"></td></tr>
<tr><td>Web of Science (Core Collection)
Platform: Web of Science</td><td>Coverage: 1965 – 22.11.2020 (12:36 CET)
Hits: 2,968</td></tr>
<tr><td colspan="2">Web of Science was searched with the following filters: Articles & English language. Moreover, the search results were limited for the categories Sports Science and Psychology.

Note: Web of Science database offers two ways for keyword search (Author Keywords & Key Words Plus). We decided to use the Author Keyword function which only searches for the keywords that were added by the authors in the original publication.

Search Strategy:
(football* OR soccer) [Title & Author Keywords]
AND
(intervention OR train* OR program* OR approach OR pract* OR effect* OR impact OR improv* OR learn* OR perform* OR coach* OR "skill acquisition" OR cognit* OR ecologic* OR constraints OR "information processing") [Title & Author Keywords]
NOT
(novice OR referee OR injur* OR pupil* OR class OR goalkeep* OR NFL OR "american football" OR “australian football") [Title & Author Keywords]</td></tr>
</table>

Note. The exported files were searched for hits in other languages than English and other publication types than journal articles. No incorrect hits were found in MEDLINE and PsycInfo. In Web of Science 15 documents were found that were not labeled as journal articles. These 15 documents were deleted manually. In SPORTDiscus, 10 items were found in other languages than English.

Supplement II: Quality in Reporting and Risk of Bias in Individual Studies

Supplement IIa: Results of the Downs & Black Checklist

Study	*Reporting*										*External Validity*			*Internal Validity - bias*							*Internal Validity - confounding (selection bias)*								*Power*		*Score* in %
	1	2	3	4	5	6	7	8	9	10	11	12	13	14	15	16	17	18	19	20	21	22	23	24	25	26	27	28	29	30	
Arslan et al., 2020	1	1	1	0	0	1	1	/	1	0	1	UD	1	/	UD	1	1	0	UD	1	1	1	1	0	0	NA	NA	1	0	0	57.69
Barquero-Ruiz et al., 2020	1	1	1	1	0	1	1	/	0	1	1	UD	UD	/	1	1	1	1	1	1	0	1	1	0	1	1	NA	UD	0	0	66.67
Bekris et al., 2012	1	1	0	0	0	1	1	/	0	1	UD	UD	UD	/	UD	1	UD	1	UD	1	1	UD	UD	0	1	UD	NA	UD	0	0	37.04
Boraczyński et al., 2019	1	1	0	1	0	1	1	/	0	1	UD	UD	1	/	UD	1	1	1	1	1	1	0	UD	0	1	UD	NA	1	0	0	55.56
Bozkurt, 2018	1	1	0	0	0	1	1	/	1	1	UD	UD	1	/	UD	1	1	1	UD	1	1	1	UD	0	0	NA	NA	1	0	0	53.85
Coutinho et al., 2018	1	1	1	0	1	1	1	/	0	NA	1	UD	1	/	UD	1	UD	1	UD	1	1	1	1	0	0	NA	0	UD	0	1	57.69
Gaspar et al., 2019	1	1	0	0	0	1	1	/	0	NA	UD	UD	1	/	0	1	1	1	UD	1	0	1	UD	0	NA	NA	NA	UD	0	1	45.83
Guilherme et al., 2015a	1	1	1	0	1	1	1	/	1	1	1	1	1	/	UD	1	1	1	UD	1	1	1	1	1	1	UD	0	1	1	0	78.57
Guilherme et al., 2015b	1	1	1	0	0	1	1	/	1	1	1	1	1	/	UD	1	1	1	UD	1	1	1	1	0	1	UD	NA	1	1	0	74.07
Haaland & Hoff, 2003	1	1	0	0	0	1	0	/	1	1	UD	UD	1	/	1	1	UD	1	UD	0	1	0	UD	0	1	UD	NA	1	0	1	48.15
Harvey et al., 2010	1	1	0	1	0	1	1	/	0	1	1	UD	1	/	UD	1	1	1	1	1	1	1	1	0	0	NA	NA	UD	0	1	65.38
Holt et al., 2012	1	1	0	1	0	1	0	/	1	NA	1	UD	UD	/	0	1	1	1	UD	1	0	1	1	0	NA	NA	NA	1	NA	NA	59.09
Hossner et al., 2016	1	1	1	0	0	1	0	/	1	1	1	UD	1	/	UD	1	1	1	UD	0	1	1	1	0	0	NA	NA	1	1	0	61.54
Kösal et al., 2020	1	1	0	0	0	1	1	/	1	1	UD	UD	UD	/	UD	1	1	0	UD	1	1	1	1	0	1	UD	NA	1	1	0	55.56
Miranda et al., 2013	1	1	0	0	0	0	1	/	1	0	1	UD	UD	/	UD	1	1	1	UD	1	0	1	UD	0	NA	NA	NA	1	0	0	44.00
Montesano & Mazzeo, 2019	1	0	0	0	0	1	0	/	1	NA	UD	UD	UD	/	UD	1	UD	1	UD	0	1	UD	UD	0	0	NA	NA	1	NA	NA	30.43
Ouzak & Çağlayan, 2019	1	1	0	0	0	1	1	/	1	1	1	UD	1	/	UD	1	1	1	UD	1	1	1	UD	0	0	NA	NA	1	0	0	57.69
Práxedes et al., 2016	1	1	0	0	0	1	1	/	1	1	1	1	UD	/	1	1	UD	1	UD	1	1	1	UD	0	0	NA	NA	1	0	0	57.69
Práxedes Pizarro et al., 2017	1	1	1	0	0	1	1	/	0	1	1	UD	UD	/	UD	1	1	1	UD	1	0	1	UD	0	NA	NA	NA	UD	0	0	48.00
Práxedes et al., 2018a	1	1	1	0	0	1	1	/	0	1	1	1	UD	/	UD	1	UD	1	UD	1	1	1	UD	0	0	NA	NA	UD	0	0	50.00
Práxedes et al., 2018b	1	1	0	1	0	1	1	/	1	1	1	UD	UD	/	UD	1	1	1	1	1	1	0	UD	0	0	NA	NA	1	0	0	57.69
Práxedes et al., 2019	1	1	0	1	0	1	1	/	0	1	1	UD	UD	/	UD	1	1	1	1	1	0	1	1	1	NA	NA	NA	UD	0	0	60.00
Raastad et al., 2016	1	1	0	1	0	1	0	/	1	1	1	UD	UD	/	UD	1	1	1	1	0	1	0	1	1	0	NA	NA	1	0	0	57.69
Radziminski et al., 2013	1	1	0	1	0	1	1	/	1	1	UD	UD	UD	/	UD	1	1	1	UD	1	1	1	UD	0	1	UD	NA	1	0	0	55.56
Roberts et al., 2020	1	1	0	1	1	1	1	/	1	1	1	1	1	/	UD	1	1	1	1	1	1	1	1	0	1	1	0	1	0	0	78.57
Santos et al., 2018	1	1	0	1	0	1	1	/	1	1	1	UD	1	/	UD	1	1	1	1	1	1	1	1	0	1	UD	NA	1	0	0	70.37
Schöllhorn et al., 2006 (study 1)	1	1	0	0	0	1	0	/	0	1	1	1	UD	/	UD	1	UD	1	UD	0	1	1	UD	0	0	NA	NA	UD	0	0	38.46
Schöllhorn et al., 2006 (study 2)	1	1	0	0	0	1	0	/	1	1	1	1	UD	/	UD	1	UD	1	UD	0	1	1	UD	1	1	UD	NA	1	0	0	51.85
Schöllhorn et al., 2012	1	1	0	0	0	1	1	/	1	0	1	UD	1	/	UD	1	1	1	UD	0	1	1	UD	1	1	1	NA	1	0	0	59.26
Schwab et al., 2019	1	1	0	1	0	1	1	/	1	1	1	1	1	/	UD	1	1	1	UD	0	1	1	UD	1	1	UD	NA	1	0	0	66.67
Sierra-Ríos et al., 2020	1	1	0	1	0	1	1	/	1	1	UD	1	UD	/	UD	1	UD	1	1	1	1	1	1	0	1	UD	NA	1	1	0	66.67
Teixeira et al., 2005	1	1	0	0	0	1	1	/	1	0	UD	UD	0	/	UD	1	1	1	UD	0	1	UD	UD	0	NA	NA	NA	1	0	0	40.00
Weigelt et al., 2000	1	1	0	0	0	1	0	/	1	0	1	UD	UD	/	UD	1	UD	1	1	0	1	0	UD	0	0	NA	NA	1	0	0	38.46
Witkowski et al., 2011	1	1	0	0	0	0	0	/	0	0	1	UD	UD	/	UD	1	1	0	UD	1	1	1	UD	0	0	NA	NA	UD	0	0	30.77
Zago et al., 2016	1	1	1	1	1	1	1	/	1	0	1	1	1	/	UD	1	1	1	1	1	1	1	1	0	1	UD	0	1	0	0	75.00
**∑**	35	34	9	13	4	33	26	/	24	24	25	10	16	/	3	35	25	32	11	25	29	27	15	6	15	3	0	25	5	4	
**Agreement R1 & R2 in %**	100	100	97.14	88.57	94.29	91.43	97.14	/	94.29	100	88.57	94.12	91.43	/	100	100	91.43	100	97.14	94.29	100	94.29	91.43	100	100	97.14	94.29	88.57	1	/	95.76
**Cohen's κ**	1	1	0.93	0.75	71.77	0.68	0.93	/	0.87	1	0.73	0.87	0.83	/	1	1	0.78	1	0.93	0.85	1	0.82	0.83	1	1	0.94	0.72	0.75	100	/	0.91

Note. "1" = yes; "0" = No; "UD" = unable to determine; "NA" = item no applicable; items 8 and 14 are not applicable for quality assessment in this systematic review.

Further explanations of item 30 (calculation of statistical power):

To examine the individual studies power (item 30), a post-hoc power analysis was conducted using G*power (version 3.1.9.6; Faul et al., 2007) to investigate whether the study provides a power of 1-β = 80 % (α = .05) to detect a moderate effect (i.e., *d* = .50) according to Cohen (1988). The statistical power was recalculated for all studies, because the five studies that conducted an a priori power analysis (see item 29) used different conventions for effect sizes, calculated the power for small or large effects, or used different α-levels. For a few non-parametric tests (e.g., Friedman test), the power cannot be calculated using G*Power. In these cases, we used correction factors referred to as the asymptotic relative efficiency (ARE) to estimate the study's power (Prajapati et al., 2010). Further, correlations among repeated measures are required to calculate the power for study designs with dependent samples. If the correlation was not reported in the original publication, conventions of Cohen (1988) for a moderate effect (i.e., *r* = .30) were used.

Two studies used magnitude-based inferences for the investigation of outcome effects (Coutinho et al., 2018; Gaspar et al., 2019). For these two studies, a spreadsheet for sample size calculation for magnitude-based inferences was used (Hopkins, 2020).

Two other studies (Holt et al., 2012; Montesano & Mazzeo, 2019) presented their results descriptively so that both items of the subscale power were rated as "not applicable" and were not considered for the calculation of the scores.

References

Cohen, J. (1988). Statistical Power Analysis for the Behavioral Sciences (2nd ed.). New York: Routledge. https://doi.org/10.4324/9780203771587

Faul, F., Erdfelder, E., Lang, A.-G., & Buchner, A. (2007). G*Power 3: A flexible statistical power analysis for the social, behavioral, and biomedical sciences. *Behavior Research Methods, 39*, 175-191.

Hopkins, W. G. (2020) Sample-size Estimation for Magnitude-Based Decisions. *Sportscience, 24*, 17-27.

Prajapati, B., Dunne, M., & Armstrong, R. (2010). Sample size estimation and statistical power analyses. *Optometry Today, 16*(7), 10-18.

Supplement IIb: Results of the TIDieR Checklist

Study	1	2	3	4	5	6	7	8	9	10	11	12	Score in %
Arslan et al., 2020	1	1	0	1	0	1	0	1	NA	NA	0	NA	55.56
Barquero-Ruiz et al., 2020	1	1	0	1	1	1	0	1	NA	NA	1	1	80.00
Bekris et al., 2012	1	0	0	0	0	0	0	0	NA	NA	0	NA	11.11
Boraczyński et al., 2019	1	1	1	1	0	1	0	1	NA	NA	1	1	80.00
Bozkurt, 2018	1	1	0	0	0	1	1	1	NA	NA	0	NA	55.56
Coutinho et al., 2018	1	1	1	0	0	1	1	1	NA	NA	0	NA	66.67
Gaspar et al., 2019	1	1	0	1	0	0	0	1	NA	NA	0	NA	44.44
Guilherme et al., 2015a	1	1	0	0	0	1	1	1	NA	NA	0	NA	55.56
Guilherme et al., 2015b	1	1	0	0	0	1	1	1	NA	NA	0	NA	55.56
Haaland & Hoff, 2003	1	1	0	0	0	1	0	0	NA	NA	0	NA	33.33
Harvey et al., 2010	1	1	0	1	1	1	1	1	NA	NA	1	1	90.00
Holt et al., 2012	1	1	1	0	1	1	0	1	1	NA	0	NA	70.00
Hossner et al., 2016	1	1	1	0	0	1	1	1	NA	NA	0	NA	66.67
Kösal et al., 2020	1	1	0	1	0	1	0	1	NA	NA	0	NA	55.56
Miranda et al., 2013	1	1	0	0	0	1	0	0	NA	NA	0	NA	33.33
Montesano & Mazzeo, 2019	0	1	1	0	0	0	0	0	NA	NA	0	NA	22.22
Ouzak & Çağlayan, 2019	1	1	1	1	0	1	0	1	NA	NA	0	NA	66.67
Práxedes et al., 2016	1	1	0	1	0	1	0	1	NA	NA	0	NA	55.56
Práxedes Pizarro et al., 2017	1	1	0	1	1	1	0	1	NA	NA	0	NA	66.67
Práxedes et al., 2018a	1	1	0	1	1	1	0	1	NA	NA	0	NA	66.67
Práxedes et al. 2018b	1	1	0	1	0	1	0	1	NA	NA	1	1	70.00
Práxedes et al. 2019	1	1	0	1	0	1	0	1	NA	NA	1	1	70.00
Raastad et al., 2016	1	1	1	1	NA	1	0	1	NA	NA	1	0	77.78
Radziminski et al., 2013	1	1	1	1	0	1	1	1	NA	NA	0	NA	77.78
Roberts et al., 2020	1	1	1	1	1	1	1	1	1	NA	1	1	100
Santos et al., 2018	1	1	1	1	0	1	1	1	NA	NA	0	NA	77.78
Schöllhorn et al., 2006 (study 1)	1	1	0	0	0	0	0	0	NA	NA	0	NA	33.33
Schöllhorn et al., 2006 (study 2)	1	1	0	0	0	0	0	1	NA	NA	0	NA	44.44
Schöllhorn et al., 2012	1	1	0	1	0	1	0	1	NA	NA	0	NA	55.56
Schwab et al., 2019	1	1	1	1	0	1	1	1	NA	NA	0	NA	77.78
Sierra-Ríos et al., 2020	1	1	0	1	1	1	0	1	NA	NA	1	0	70.00
Teixeira et al., 2003	1	1	0	0	0	1	0	1	NA	NA	0	NA	44.44
Weigelt et al., 2000	1	1	1	0	NA	0	0	1	NA	NA	0	NA	50.00
Witkowski et al., 2011	0	1	0	0	0	0	0	0	NA	NA	0	NA	11.11
Zago et al., 2016	1	1	1	1	1	1	1	1	NA	NA	1	1	100
Σ	33	34	13	20	8	28	11	29	2	0	9	7	
**Agreement R1 & R2 in %**	100	100	94.29	94.29	100	94.29	94.29	94.29	100	/	88.57	97.14	96.43
**Cohen's κ**	1	1	0.87	0.88	1	0.8	0.87	0.82	1	/	0.74	0.65	0.92

Supplement III: Detailed Description of Intervention Content and Methods

Supplement IIIa: Detailed description of the practice content and methods from studies investigating theory-driven instructional approaches

**Author (year)**	**Practice content and delivery**		**Methods**		
	Experimental group	Other groups (e.g., CGs)	Measurements	Instruments	Reliability
**Differential Learning (DL; *n* = 9)**					
Bozkurt (2018)	The DL group practiced nine target exercises that aimed at improving passing, dribbling, and feet-juggling. The exercises included high inter-trial variability through movement variations in the standing or kicking leg, arms, trunk, head, as well as ball. The techniques were practiced in blocked order. No corrective feedback was delivered.	TL focused on ideal movement archetypes. Nine exercises were practiced for target-passing, dribbling, and feet-juggling, respectively. Techniques were practiced in blocked order. Numerous repetitions were conducted, and error-based corrections were delivered by a coach.	1. pre-test<br>2. 4-week post-test	*Skill tests:*<br>-Mor-Christian soccer test (Passing)<br>-German Football Association test (dribbling, agility, and juggling)	yes<br>(but reference is missing in the reference list)
Coutinho et al. (2018)	The DL groups' practice consisted of physical literacy combined with technical exercises as well as SSGs. All exercises were grounded in a high movement variability and unpredictability.	The CGs participated in their usual team practice sessions and did not receive an enrichment program.	1. pre-test<br>2. 10-week post-test	*Syst. Observation:*<br>-technical performance observation<br>-CBATs (5v5 + GK)	yes<br>(Santos et al., 2017)<br><br>Syst. Observation: intra-RR: ICC < .88
Gaspar et al. (2019)	*Differential Learning protocol:* DL in a blocked order was conducted to improve kicking accuracy. Participants kicked the ball by applying a high variability in their movement (18 variations). No corrective feedback was delivered. The players kicked 36 shots from three different positions. Six shots were made static and after short dribblings. *Traditional training protocol*: Thirty-six kicking repetitions in blocked order from three positions were made. The participants were	No other groups were included.	1. baseline<br>2. post-test<br>3. post-test after a match lasting 35 min.	*Skill tests:*<br>-shooting accuracy and speed	yes<br>(Markovic et al., 2006)

	encouraged to kick the ball with maximum accuracy and speed. Corrective feedback, focusing on error description, movement-oriented correction, and metaphoric instruction was delivered.				
Hossner et al. (2016)	*Differential Learning:* The program aimed at improving the goal-shot precision and included 13 sources of variation focusing on the supporting leg, the kicking foot, as well as the trunk position. Moreover, movement variants regarding the kicking movement and ball characteristics were used. No instructions were delivered.<br>*Differential Learning & Feedback:* The protocol was similar to the DL group but additional augmented feedback by an instructor was delivered.	The CG practiced according to TL methods. The practice consisted of methodologically structured practice exercises to improve goal-shot precision, following principles such as "from easy to complex". Exercises aimed at improving the sweet-spot kick, the inside and outside kick. Three to four exercises were conducted per session, each lasting eight to ten minutes. The participants received augmented feedback from an instructor based on an ideal movement technique.	1. pre-test<br>2. 8-week post-test<br>3. 1-week ret-test	*Skill test:*<br>-shooting accuracy test	no
Ozuak & Çağlayan (2019)	After a "standard" warm-up of 15 minutes, the DL group participated in exercises that were characterized by a high inter-trial variability through random perturbations (e.g., different movements or changes in equipment). Coaches' feedback was reduced to a minimum.	The CG participated in their usual practice that was not further specified.	1. pre-test<br>2. 8-week post-test	*Skill test:*<br>-creative speed test<br>-ball dribbling test<br>-ball juggling test<br>-passing test	yes<br>(Bangsbo & Mohr, 2011; Bradford & Strand, 1993; Sualp, 2018)
Santos et al. (2018)	DL was conducted at the beginning of the players' team practice sessions and aimed at improving the in-game creativity. The practice program consisted of SSGs in a random order (e.g., 1v2; 3v3; 1v1), focusing on intertrial variability. No error corrections were delivered.	Game-based practice within different SSGs was delivered according to methodological principles, such as 'from easy to complex'. Specific instructions and corrections were delivered.	1. pre-test<br>2. 20-week post-test	*Syst. Observation:*<br>-CBATS<br>-positional data<br>(5v5 + GK)	yes<br>(Santos et al., 2017)<br><br>intra-RR:<br>ICC > .81

Schöllhorn et al. (2006; study 1)	The IG participated in a supplemental DL program to improve players' dribbling and passing skills. No specifications of the practice program were provided but authors referred to methods reported by Schöllhorn (1999).	TL group participated in practice sessions with detailed descriptions of an ideal movement technique for slalom dribbling and passing. A high number of repetitions and many corrective instructions were delivered by a coach.	1. pre-test<br>2. 4-week post-test	*Skill test:*<br>-dribbling & passing complex test	no
Schöllhorn et al. (2006; study 2)	The additional practice of the experimental group was based on the principles of DL and aimed at improving players' shooting skills. The practice was characterized by no specific repetitions. The coaches did not deliver any corrective or error-based feedback.	The TL group practiced according to conventional methods associated with chip shot including a high number of repetitions and corrective feedback (error description, movement-oriented correction, & metaphoric instructions) after each shot.	1. pre-test<br>2. 6-week post-test<br>3. 1-year ret-test	*Skill test:*<br>-goal shooting accuracy test	no
Schöllhorn et al. (2012)	*Differential Learning (blocked):* Practice of goal shot and ball control was delivered according to principles of DL (high number of variations of the movement) in blocked order without corrective feedback. The practice aimed to seek and explore functional movement patterns.<br>*Differential Learning (random)*: Same practice content as in the blocked group but the practice was conducted in random order.	The CG practiced the goal shot and ball control technique by using a "classical" TL approach focusing on an ideal movement technique. Both techniques were practiced in blocks (first shooting, then ball control). Error corrections in form of instructions were delivered by a coach.	1. pre-test<br>2. 4-week post-test<br>3. ret-test	*Skill tests:*<br>-goal-shooting accuracy<br>-ball reception test	no
**Teaching Games for Understanding (TGFU; *n* = 5)**					
Barquero-Ruiz et al. (2020)	Two practice groups participated in a TGFU intervention, led by trained coaches. The intervention aimed at improving offensive and defensive principles of play. Each session started with a game form (20 min.). Then, a physically inactive reflection phase ("teaching for understanding"; 5-7 min.) was applied followed by technical drill practice (15 min.). After that, another game form	No other groups were included.	1. pre-test<br>2. 4-week post-test	*Syst. Observation:*<br>-GPAI (5v5)	yes<br>(Oslin et al., 1988)<br><br>inter-RR:<br>81.5-93.7 %<br>ICC < .93

	(25 min.) was conducted, before "review and closure" (5-7 min.).				
Harvey et al. (2010)	A similar practice program for both the varsity and first-year teams was conducted: The TGFU intervention aimed at developing the players in "defending as a unit of three players". The sessions focused on defensive off-the-ball aspects. All sessions related to the tactical problem of "preventing scoring by defending space in the field". SSGs, phases of play (e.g., attack vs. defense), and functional practice (e.g., game that ascertains technical and tactical abilities) were applied.	No other groups were included.	1. baseline (4 sessions)<br>2. int-1 (1 session)<br>3. int-2 (1 session)<br>4. int-3 (1 session)	*Syst. Observation:*<br>-GPAI (3v3 + GK for the defending team)	yes<br>(García López et al., 2013)<br><br>inter-RR: < 80 %
Práxedes et al. (2016)	The IG participated in a TGFU program that consisted of modified SSGs. Four tasks, each lasting 15 minutes, were carried out that focused on one tactical principle in the attack (e.g., keeping possession of the ball). Task constraints were grounded in the pedagogical principles of representation and exaggeration. The coach acted as a facilitator by using a question-and-answer approach.	The CG participated in TL with the same structure of 4x15 minute exercises as in the IG but based on a technical approach. Explicative and prescriptive instructions were used by the coach.	1. pre-test (3 matches)<br>2. post-test (3 matches)	*Syst. Observation:*<br>-GPET (n. r.)	yes<br>(García López et al., 2013)<br><br>intra-RR: κ > .90<br>time reliability analysis: κ > .85
Práxedes Pizarro et al. (2017)	The intervention program followed the methodological principles of TGFU. In each session, four 15-minute playing forms were conducted where determining factors (e.g., number of players) were modified. The trained coach applied a question-and-answer approach to coaching. The complexity of the practice program increased progressively.	No other groups were included.	1. pre-test<br>2. int-test (6 matches)<br>3. final test (5 matches)	*Syst. Observation:*<br>-GPET (7v7)	yes<br>(García López et al., 2013)<br><br>intra-RR: κ > .90<br>time reliability analysis: κ > .85

Sierra-Ríos et al. (2020)	A comprehensive teaching program based on principles of TGFU was delivered by a trained coach. SSGs were conducted after a playful warm-up. In an initial reflection, the objective of the session was introduced. The sessions consisted of modified games where the coach applied a question-and-answer approach focusing on the tactical aim of the sessions (e.g., generate free spaces or progression by numerical superiority).	The CG participated in a practice program based on direct instruction following the same structure as the TGFU group: warm-up (continuous running & mobility), technical/analytical exercises, a game, and, finally, stretching exercises. The coach used a direct instructional approach to achieve the session's objectives (e.g., create combined actions with passes or dribbling).	1. pre-test<br>2. 6-week post-test	*Syst. Observation:*<br>-GPET (n. r.)	yes<br>(García López et al., 2013)<br><br>reliability: α = .75
**Non-linear pedagogy (NLP; *n* = 4)**					
Práxedes et al. (2018a)	The practice program was grounded on principles of NLP by using modified games and numerical superiority in the attack. All exercises are aimed at achieving tactical offensive and defensive objectives. Every session consisted of four 15-minute exercises and referred to a principle of play.	The CG participated in a practice program that was developed based on a TL approach, prioritizing technical components. The coach applied a direct instructional approach.	1. pre-intervention (3 matches)<br>2. intervention (7 matches)	*Syst. Observation:*<br>-GPET (8v8)	yes<br>(García López et al., 2013)<br><br>inter-RR: κ <.90<br>intra-RR: κ <.92
Práxedes et al. (2018b)	The same protocols were used for both the low- and average performance groups.<br>*Intervention 1:* A practice program based on principles of NLP by using SSGs with numerical superiority in the attack was delivered. The principles representation, tactical complexity, and exaggeration were applied.<br>*Intervention 2:* The practice program also followed the principles of NLP but using modified games with numerical equality.	No other groups were included.	1. pre-intervention 1 (3 matches)<br>2. intervention 1 (7 matches)<br>3. pre-intervention 2 (3 matches)<br>4. intervention 2 (7 matches)	*Syst. Observation:*<br>-GPET (n. r.)	yes<br>(García López et al., 2013)<br><br>inter-RR:<br>agreement > 90 %<br>κ >.81
Práxedes et al. (2019)	The practice program of the IGs based on the principles of NLP.	No other groups were included.	1. pre-intervention (3 matches)	*Syst. Observation:*<br>-GPET (7v7)	yes

	Each session referred to an offensive or defensive principle of play (e.g., maintain possession of the ball or preventing passing lines). SSCGs (2 to 5 players a-side) with numerical superiority at one side were used.		2. intermediate (3 matches)<br>3. final (4 matches)<br>4. retention (3 matches)		(García López et al., 2013)<br><br>inter-RR: κ > .90<br>intra-RR: κ > .92
Roberts et al. (2019)	Practice aimed at improving players' individual learning objectives by using a NLP approach, integrating technical and tactical skills. A 'representative learning design' was chosen by using predominantly playing form activities to create perception-action couplings and promote self-organization processes. The role of the full-time professional and trained youth academy coach was to manipulate task constraints to refine the practice activities to the players' requirements.	The practice of the control period corresponded to a linear approach, based on information-processing theory. The full-time professional youth academy coach acted as an expert who aimed at fostering predetermined outcomes. A controlled and predictable environment, by using predominantly training form activities and high volumes of instructions, was created.	1. pre-intervention<br>2. 8-week post-test	*Skill test:*<br>-LSST	yes<br>(Ali et al., 2007)

Note. CG = control group; DL = Differential Learning; ICC = intraclass correlation coefficient; TL = Traditional Learning; LSST = Loughborough Shooting Skill Test; RR = rater-reliability.

Supplement IIIb: Detailed description of the practice content and methods from studies investigating specific aspects of practice or coaching

Author (year)	Practice content and delivery		Methods		
	Experimental group	Other groups (e.g., CGs)	Measurements	Instruments	Reliability
**Effects of technical drill practice (with subsequent SSGs or coordination exercises; *n* = 7)**					
Boraczyński et al. (2019)	The practice sessions of the proprioceptive-coordination group (PCT) aimed at improving generic and specialized coordination skills, speed, and individual soccer-specific technique through targeted drills and SSCGs (e.g., 3v3 or 5v5). Before the sessions, the proprioceptive-coordinative group additionally received a series of 24 multimode proprioceptive exercises where soccer-specific techniques were combined with proprioceptive-coordination elements.	*Regular training group:* The sessions are aimed at improving generic and specialized coordination skills, speed, and individual technique through targeted drills and SSCGs (e.g., 3v3 or 5v5). Instead of the PC-T exercises, the regular training group participated in SSCGs.<br><br>*Control group*: No soccer-specific practice.	1. pre-test<br>2. 6 months peri-training<br>3. 6-months post-training	*Skill tests:*<br>-Movement rhythm test<br>-Motor adaption test<br>-Static balance test<br>-Kinaesthetic differentiation test	yes<br><br>(relative reliability: .59 ≤ ICC ≤ .77)
Holt et al. (2012)	After seven baseline practices of the "passing square" the players participated in five sessions for improving awareness (i.e., over shoulder glance to check position and movement of the support player at the next cone), six sessions for passing, and four sessions for first touch skills. Two practices of the respective skills were included in each session and were immediately followed by a 4v4 SSG. The intervention consisted of peer-assessed feedback, goal setting, and group contingency. The players self-set goals regarding a percentage of correct executions they would like to achieve during the intervention.	No other groups were included.	1. Baseline (7 sessions)<br>2. Awareness (5 sessions)<br>3. Passing (6 sessions)<br>4. First touch (4 sessions)	*Syst. Observation:*<br>-predetermined technical criteria (technical drill)	inter-RR:<br>overall IRA:<br>83.4 %<br>mean IRA:<br>87.8 %
Kösal et al. (2020)	The sessions of the coordination group included a warm-up (10 min.), football-specific exercises (50 min.),	*Control group regular training:* The sessions included a warm-up (10 min.), football-specific	1. pre-test<br>2. 10-week post-test	*Skill tests:*<br>-Mor & Christian test battery	yes

	and cooling (15 min.). Additionally, the players participated in a specific coordination program lasting for 30 min., combining coordination and soccer-specific technical exercises.	exercises (50 min.), and cooling (15 min.). Instead of coordination exercises, the players participated in their usual care practice. *Control group without coach-led training:* The players participated in soccer-specific practice that was not led by coaches and did not follow a specific protocol.		-Yeagley's Test<br>Johnson football skill test	(Mor & Christian, 1979; Vanderford et al., 2004)
Miranda et al. (2013)	The players of the IG participated in a soccer-specific positional practice as well as general technical-tactical exercises (e.g., the attack against the defence in reduced fields) and simulated matches.	No other groups were included.	1. pre-test<br>2. post-test	*Skill tests:*<br>-zigzag dribbling test<br>-lob pass test<br>-juggling test	yes<br>(VanRosum & Wijbena, 1991; Vaeyens et al., 2006)
Montesano & Mazzeo (2019)	The players of the IG participated in additional practice sessions to improve technical and physical characteristics. The sessions started with mobilization exercises followed by real game situations. The additional exercises included strengthening and continuous technical exercises.	The same protocol as in the IG except for the additional exercises was applied.	1. initial test<br>2. intermediate test<br>3. final test	*Skill tests:*<br>-passing precision<br>-goal shooting accuracy	no
Weigelt et al. (2000)	The IG participated in an additional practice program where feet juggling was practiced. The participants did not receive any guidance or feedback.	The CG did not participate in any additional practice.	1. pre-test<br>2. 4-week post-test	*Skill tests:*<br>-juggling test<br>-ball reception test (strong & weak foot)	no
Zago et al. (2016)	The IG participated in a practice program where tape matrix structures as three-dimensional spatio constraints were used for identifying specific areas on the ground. By using these constraints, analytical/technical drills, and phases of play games (e.g., 1v1 or 2v1) were conducted.	The CG participated in a practice program with the same content but without three-dimensional spatio constraints. The sessions included a warm-up, soccer-specific technical drills, situation games, and SSGs. The practice followed methodological recommendations of the Italian Football Associations.	1. pre-test<br>2. 22-week post-test	*Skill tests:*<br>-LSPT<br>-shuttle Dribble Test<br>-slalom Dribble Test	yes<br>(Ali et al., 2007; Lemmink et al., 2004)

**Practice to reduce lateral asymmetries or improve the non-dominant leg performance (*n* = 5)**					
Guilherme et al. (2015a)	The additional non-dominant leg practice included technical exercises of specific motor skills (i.e., receiving, passing, driving, dribbling, shooting, and disarm/interception). The drill-based practice was conducted during the first 20 minutes of the team practice sessions.	While one group participated in the intervention program, the other group did not participate in any additional practice.	1. baseline<br>2. 4-month assessment<br>3. 8-month assessment	*Syst. Observation:*<br>-SAFALL-FOOT (5v5)	yes (Guilherme et al., 2012)<br><br>(no IRR proofed in the study)
Guilherme et al. (2015b)	The IG participated in a technical practice program to improve soccer-specific motor skills (i.e., receiving, passing, driving, dribbling, shooting, disarm/interception) focusing on the non-preferred leg. Practice was conducted during the first 20 minutes of the team session and only included technical drills.	The CG participated in practice that does not include special exercises of the lower limbs and that does not include any guidance.	1. pre-test<br>2. 6-months post-test	*Syst. Observation:*<br>-SAFALL-FOOT (5v5)	yes (Guilherme et al., 2012)<br><br>(no IRR proofed in the study)
Haaland & Hoff (2003)	The IG participated in an increased volume of soccer practice with the left, non-preferred leg. No specific practice was conducted but the players only used their left leg in all practice parts, except full play.	The CG participated in all practice sessions by using both legs and no other restrictions.	1. pre-test<br>2. 8-week post-test	*Skill tests:*<br>-slalom dribble test<br>-receiving & direct volley shot test<br>-one-touch passing test	no
Teixeira et al. (2003)	The IG used in three out of five weekly sessions only the non-preferred leg in technical drills as well as small games (45 min. per session). During the intervention period, the complexity of the tasks was increased regarding the accuracy and force in the kicks, the control and speed in the dribbling tasks, as well as by combining these basic skills into serial motor tasks.	Players that were not assigned to the IG participated in all sessions by using both feet.	1. pre-test<br>2. 4-month post-test	*Skill tests:*<br>-speed of dribbling<br>-kicking for accuracy<br>-kicking for force	no
Witkowski et al. (2011)	*Experimental group 1:* The players participated in a practice program where the non-dominant leg was predominantly used in technical	The players in the CG participated in a regular practice without specifications regarding the use of the dominant or non-dominant leg.	1. pre-test<br>2. 6-month int-test<br>3. 6 month-post-test	*Skill tests:*<br>-test battery of Witkowski & Ljach (2006)	yes (Witkowski & Ljach, 2006)

	exercises.<br>*Experimental group 2:* The players participated in a practice program where both the dominant and non-dominant leg were trained equally.				
**Effects of game-base practice programs (*n* = 2)**					
Arslan et al. (2020)	*SSG group:* The players participated in a practice program based on SSGs. SSGs were applied by using small goals, floater players, as well as SSGs with goalkeepers. At the end of the sessions, 11v11 matches were conducted.	*HIIT group:* After the warm-up, the players participated in a High Intensity Interval Training (HIIT) program. At the end of the sessions, 11v11 matches were conducted.	1. pre-test<br>2. 8-week post-test	*Skill tests:*<br>-zigzag agility with ball<br>-speed dribbling test	yes<br>(Little et al., 2005; Taşkin, 2008)
Radziminski et al. (2013)	*SSG group:* The players participated in 3v3 SSGs that were applied with modifications, for example through the use of floater players. In each session, 5 games á 4 min. were conducted.	*Running group:* The players conducted an interval training program, including high-intensity exercises.	1. pre-test<br>2. 16-week post-test	*Skill tests:*<br>-juggling<br>-rotation pass<br>-passing<br>-dribbling<br>-heading<br>-bench passing<br>-shooting accuracy	yes<br>(test of the German Football Association but reference in the reference list is missing)
**Practice with modified ball sizes (*n* = 2)**					
Bekris et al. (2012)	*Competitive group:* Practising with size-2 ball coordination and game situations.<br>*20-Min group*: Practising 20-minute sessions with a size-2 ball (no specification of the practice protocol was made).<br>*30-min group*: Practising 30-minute sessions with a size-2 ball (no specification of the practice protocol was made).	*Control group: Same* content as the 20-Min group by using a size 4-ball.	1. pre-test<br>2. post-test	*Skill tests:*<br>-Passing<br>-Juggling<br>-Running with the ball<br>-Turns	yes<br>(Rosch et al., 2000; Russel, 1991; Vale et al., 2009)
Raastad et al. (2016)	Both groups (i.e., smaller & larger ball groups) participated in a practice program where only soccer juggling with the respective smaller or larger ball was conducted. No	No other groups were included.	1. pre-test<br>2. 6-week post-test<br>3. 6- to 7-week ret-test	*Skill tests:*<br>-ball juggling<br>-ball reception test	Juggling: no<br>Ball reception: yes<br><br>(Retest-R.: .98 ≤ ICC ≤ .99)

	instructions or feedback were delivered to any of the groups.				
**Internal and external focus feedback (*n* = 1)**					
Schwab et al. (2019)	*External group:* Participants practiced the knuckle ball free kick technique and received external focus feedback after every third shot. The practice was conducted before the team sessions. Instructions focused on the movement effects, for example, on the ball velocity or movement of the ball (i.e., "Make sure to hit the ball just below its midline to lift the ball").	*Internal group:* Participants practiced the knuckle ball freekick technique and received internal focus feedback (i.e., focus on the movement themselves) after every third shot. The practice sessions were conducted before the team sessions. Instructions focused on the movement (i.e., "concentrate on hitting the ball exactly with the inner side of your foot").	1. pre-test<br>2. 5-week post-test<br>3. 3-week ret-test	*Skill test:*<br>-Adidas miCoach smart ball	no

Note. CG = control group; DL = Differential Learning; IG = intervention group; TL = Traditional Learning; RR = rater-reliability; SAFALL-FOOT = System of Assessment of Functional Asymmetry of the Lower Limbs in Football; succ. = successful

Supplement IV: Narrative Summary of Results from Studies without a Control Group

Author (year)	Groups	Intervention duration		Statistical Analysis	Outcome variables	Main results
		Sessions (min.)	Weeks			
Gaspar et al. (2019)[a,]	-all players (*n* = 20) participated in one session DL and one session TL	20 (25)	10	Non-clinical magnitude-based inferences	*Skill-test:*<br>-shooting velocity<br>-shooting accuracy	Better acute effects after DL compared to the TL protocol were found regarding a small increase in shooting velocity. DL did not impact the overall shooting accuracy, although slightly better accuracy in the higher scoring zones was found.
Barquero-Ruiz et al. (2020)[a]	-TGFI 1 (*n* = 10)<br>-TGFU 2 (*n* = 10)	6 (90)	1	t-tests	*In-game (on-the-ball):*<br>-decision-making<br>-skill execution<br>-succ. game performance<br>-number of decisions made<br>-game involvement	The number of decisions made as well as the overall game involvement increased from pre- to post-test. Improvements in the players' decision-making, skill execution, and successful game performance were found.
Harvey et al. (2010)	-first year (*n* = 18)<br>-varsity (*n* = 18)	8 (45-60)	12	t-tests	*In-game:*<br>-decision-making<br>(on- and off-the-ball)<br>-skill execution<br>(on- and off-the-ball)<br>-adjust (off-the-ball)<br>-cover (off-the-ball)	Improvements from baseline to last assessment in both teams were found regarding appropriate adjusts. First-year players' appropriate covers, efficient skill execution, overall game involvement, and successful game performance increased. In the varsity team, more inappropriate covers were found at post-test. Game involvement remained equal but an increase in unsuccessful actions was found.
Práxedes Pizarro et al. (2017)	-TGFU (*n* = 9)	22 (60)	12	Friedmann	*In-game:*<br>-decision-making<br>(passing & dribbling)<br>-skill execution<br>(passing & dribbling)	Significant improvements were found for the decisions and executions in passing as well as for the executions in dribbling. No improvements regarding decisions to dribble were found. Improvements were also found in the total game performance in decision-making and skill execution. Significant differences were only found when the whole intervention was completed.
Práxedes et al. (2018b)	-low skill (*n* = 9)<br>-average skill (*n* = 10)	Int. 1:<br>14 (60)	2 x 7	Repeated measures MANOVA	*In-game:*<br>-decision making of passes<br>-skill execution of passes	In average-skill players, the numerical superiority intervention had a positive effect on passing decisions and executions. No improvements in the low-skill group were found. No group

		Int. 2:<br>14 (60)				improved in the numerical equality intervention. Improvements in low-skill players were found in passing executions after both interventions.
Práxedes et al. (2019)	-NLP (n = 19)	14 (60)	7	Friedmann & Wilcoxon	*In-game:*<br>-decision making (passes)<br>-skill execution (passes)	The decision-making and execution performance of the players was significantly better at the intermediate and final measurements compared to pre-intervention. Significantly higher performance at the ret-test compared to the pre-test, but no differences compared to the final measurement, were found.
Holt et al. (2012)	-intervention (n = 5)	22 (120)	11	Descriptive analysis	*Systematic observation of performance in a drill:*<br>-awareness<br>-passing<br>-first touch<br>(each for the strong and weak foot)	All participants improved in awareness, passing, and first touch skills. The performance was more consistent after the intervention so that the individual goals of the players were achieved. The baseline performance of the players as well as the learning rates during the intervention differed between the players. The players' performance after the intervention was maintained in most cases.
Miranda et al. (2013)	-intervention (n = 13)	n. r. (n. r.)	10	t-tests	*Skill tests:*<br>-slalom dribble<br>-lob pass<br>-juggling	Significant improvements after the intervention were found in the players' slalom dribbling and lob pass performance. No improvements in juggling were found.

[a] Further soccer-unspecific assessments (e.g., physiological tests) were conducted

Supplement V: PRISMA Checklist

Section/topic	#	Checklist item	Reported on page #
**TITLE**			
Title	1	Identify the report as a systematic review, meta-analysis, or both.	1
**ABSTRACT**			
Structured summary	2	Provide a structured summary including, as applicable: background; objectives; data sources; study eligibility criteria, participants, and interventions; study appraisal and synthesis methods; results; limitations; conclusions and implications of key findings; systematic review registration number.	1-2
**INTRODUCTION**			
Rationale	3	Describe the rationale for the review in the context of what is already known.	3
Objectives	4	Provide an explicit statement of questions being addressed with reference to participants, interventions, comparisons, outcomes, and study design (PICOS).	4
**METHODS**			
Protocol and registration	5	Indicate if a review protocol exists, if and where it can be accessed (e.g., Web address), and, if available, provide registration information including registration number.	4
Eligibility criteria	6	Specify study characteristics (e.g., PICOS, length of follow-up) and report characteristics (e.g., years considered, language, publication status) used as criteria for eligibility, giving rationale.	4; Table 1
Information sources	7	Describe all information sources (e.g., databases with dates of coverage, contact with study authors to identify additional studies) in the search and date last searched.	4 & Supplement 1
Search	8	Present full electronic search strategy for at least one database, including any limits used, such that it could be repeated.	4 & Supplement 1
Study selection	9	State the process for selecting studies (i.e., screening, eligibility, included in systematic review, and, if applicable, included in the meta-analysis).	4-5; Figure 1
Data collection process	10	Describe method of data extraction from reports (e.g., piloted forms, independently, in duplicate) and any processes for obtaining and confirming data from investigators.	5
Data items	11	List and define all variables for which data were sought (e.g., PICOS, funding sources) and any assumptions and simplifications made.	5-6; Table 1

Risk of bias in individual studies	12	Describe methods used for assessing risk of bias of individual studies (including specification of whether this was done at the study or outcome level), and how this information is to be used in any data synthesis.	5-6
Summary measures	13	State the principal summary measures (e.g., risk ratio, difference in means).	6
Synthesis of results	14	Describe the methods of handling data and combining results of studies, if done, including measures of consistency (e.g., I^2) for each meta-analysis.	6
Risk of bias across studies	15	Specify any assessment of risk of bias that may affect the cumulative evidence (e.g., publication bias, selective reporting within studies).	/
Additional analyses	16	Describe methods of additional analyses (e.g., sensitivity or subgroup analyses, meta-regression), if done, indicating which were pre-specified.	/
**RESULTS**			
Study selection	17	Give numbers of studies screened, assessed for eligibility, and included in the review, with reasons for exclusions at each stage, ideally with a flow diagram.	5 (Figure 1)
Study characteristics	18	For each study, present characteristics for which data were extracted (e.g., study size, PICOS, follow-up period) and provide the citations.	Tables 2 & 3; Supplement 3
Risk of bias within studies	19	Present data on risk of bias of each study and, if available, any outcome level assessment (see item 12).	12-13; Figure 2; Supplement 2
Results of individual studies	20	For all outcomes considered (benefits or harms), present, for each study: (a) simple summary data for each intervention group (b) effect estimates and confidence intervals, ideally with a forest plot.	12-14; Tables 5 & 6; Supplement IV
Synthesis of results	21	Present results of each meta-analysis done, including confidence intervals and measures of consistency.	/
Risk of bias across studies	22	Present results of any assessment of risk of bias across studies (see Item 15).	/
Additional analysis	23	Give results of additional analyses, if done (e.g., sensitivity or subgroup analyses, meta-regression [see Item 16]).	/
**DISCUSSION**			
Summary of evidence	24	Summarize the main findings including the strength of evidence for each main outcome; consider their relevance to key groups (e.g., healthcare providers, users, and policy makers).	14
Limitations	25	Discuss limitations at study and outcome level (e.g., risk of bias), and at review-level (e.g., incomplete retrieval of identified research, reporting bias).	20-23

Conclusions	26	Provide a general interpretation of the results in the context of other evidence, and implications for future research.	22-23
**FUNDING**			
Funding	27	Describe sources of funding for the systematic review and other support (e.g., supply of data); role of funders for the systematic review.	23

Note. All pages refer to the published article.

Reference

Moher D., Liberati A., Tetzlaff J., Altman D. G., The PRISMA Group (2009). Preferred Reporting Items for Systematic Reviews and Meta-Analyses: The PRISMA Statement. *PLoS Medicine* 6(7): e1000097. https://doi.org/10.1371/journal.pmed.1000097
